# Comparative transcriptomics of an arctic foundation species, tussock cottongrass (*Eriophorum vaginatum*), during an extreme heat event

**DOI:** 10.1038/s41598-020-65693-8

**Published:** 2020-06-02

**Authors:** Jonathon E. Mohl, Ned Fetcher, Elizabeth Stunz, Jianwu Tang, Michael L. Moody

**Affiliations:** 1 Bioinformatics Program, University of Texas at El Paso, El Paso, TX 79968 USA; 2 Institute for Environmental Science and Sustainability, Wilkes University, Wilkes-Barre, PA 18766 USA; 3 Biological Sciences, University of Texas at El Paso, El Paso, TX 79968 USA; 4 The Ecosystems Center, Marine Biological Laboratory, Woods Hole, MA 02543 USA

**Keywords:** Transcriptomics, Plant genetics, Molecular ecology, Evolution, Genetics, Plant sciences

## Abstract

Tussock cottongrass (*Eriophorum vaginatum*) is a foundation species for much of the arctic moist acidic tundra, which is currently experiencing extreme effects of climate change. The Arctic is facing higher summer temperatures and extreme weather events are becoming more common. We used Illumina RNA-Seq to analyse cDNA libraries for differential expression of genes from leaves of ecologically well-characterized ecotypes of tussock cottongrass found along a latitudinal gradient in the Alaskan Arctic and transplanted into a common garden. Plant sampling was performed on a typical summer day and during an extreme heat event. We obtained a *de novo* assembly that contained 423,353 unigenes. There were 363 unigenes up-regulated and 1,117 down-regulated among all ecotypes examined during the extreme heat event. Of these, 26 HSP unigenes had >log2-fold up-regulation. Several TFs associated with heat stress in previous studies were identified that had >log2-fold up- or down-regulation during the extreme heat event (e.g., DREB, NAC). There was consistent variation in DEGs among ecotypes, but not specifically related to whether plants originated from taiga or tundra ecosystems. As the climate changes it is essential to determine ecotypic diversity at the genomic level, especially for widespread species that impact ecosystem function.

## Introduction

The genetic mechanisms underlying local adaptation are a major focus of molecular ecology in the genomics age^1,2^. Local adaptation is well documented for many plant taxa that show variation in phenotype attributable to abiotic factors such as precipitation and temperature as well as biotic ones such as herbivory and parasitism^3^. Technological advances in transcriptomics have made it possible to use next generation sequencing (NGS) and RNA-Seq methods to identify differentially expressed genes (DEGs) between species^4,5^ and populations^6,7^ with the goal of identifying genes or functional groups of genes important for adaptation. There have been incredible advances using model organisms for the recognition of genes that may be important in pathways of adaptation to different environments^8–10^. These advances now make it possible to improve our understanding of non-model species^4,11,12^.

Transcriptomics has great potential for examining effects of climate change on plants, as it can sample many genes simultaneously and recognise otherwise cryptic physiological responses^13,14^. Under a warming climate, genetic responses to heat stress and drought are of particular interest. Studies using model organisms have recognised heat shock proteins (HSPs) as well as transcription factors (TFs) that are responsive to abiotic stress^11,15,16^. Recent research on non-model organisms has focused on crop and forestry species that have been exposed to controlled heat stress. For example, several classes of HSPs were up-regulated when spinach was exposed to heat stress, along with differential responses for TFs such as heat shock factors (HSFs) and dehydration responsive element binding proteins (DREB)^17^. Similarly, *Abies koreana* was grown under heat stress and differential expression of hundreds of HSPs and TFs were identified^18^.

The arctic tundra ecosystem is facing some of the most dramatic effects of climate change with current models suggesting an increase of up to 11°C in temperature by 2100^19,20^. These changes are leading to range shifts of many taxa^21^. In Alaska, the climate optima for ecotypes of the dominant tundra tussock cottongrass, *Eriophorum vaginatum* (Cyperaceae), have already been displaced 140 km northwards^22^. It is a foundation species throughout the moist acidic arctic tundra, where it can account for up to one‐third of ecosystem productivity^23^ and is a model for understanding local adaptation in the face of climate change^24^ because of the variation across its latitudinal range in annual temperature, precipitation, day length, and permafrost depth^22^.

Populations of *E. vaginatum* show measurable phenotypic variation across a latitudinal environmental gradient from 65°N to 70°N, much of which is retained when plants from different latitudes are grown together in common gardens, as has been described from long term ecological studies^24–27^. For example, cottongrass tussocks that were transplanted back into their home-site gardens had generally higher survival rates, flower production, and biomass than plants from “away” sites^27^, whereas light-saturated photosynthetic rate and stomatal density were correlated with latitude of population origin^27,28^. In most cases differences in long term survival and plastic responses were also associated with whether the site of origin was north or south of the treeline^27–29^. Because of these studies and the recognition of the important role that *E. vaginatum* has in ecosystem function^25^, it has been recommended as a model system for genomic sequencing to understand genetic mechanisms for adaptation to arctic environments^30^.

Because variation in ecotypic responses are measurable through field studies, transcriptomics should provide empirical evidence of the genes that have a potential role in ecotypic variation and adaptation while uncovering cryptic variation^31–33^. Genes involved in abiotic stress response and metabolic processes would be expected to show variation in expression associated with *E*. *vaginatum* ecotypes that go beyond field measurable responses^27–29^. Experimental research in common gardens has already shown significant differences in gene expression related to the home-site environment of different ecotypes^6,34,35^, especially for genes related to abiotic stress response^11,17,36^. Understanding performance of ecotypes of widespread species at the level of gene expression can provide insight as to how foundation species, which have a strong influence on ecosystem structure and function^37^, are effected by climate shifts across their geographic range. Gene expression research for ecotypes response under abiotic stress can be particularly informative in common gardens, as environmental variables that could affect genetic response in a natural setting are present^38–40^. Understanding plant response during extreme events in a field setting can be particularly valuable, but also logistically challenging, thus these studies are rare. Here, field site monitoring provided a rare opportunity for sampling on a day of extreme temperatures in a common garden in the Arctic.

Here, we combine the knowledge of ecotypic variation and transcriptomics to identify genes that may play a role in adaptations important for plants to prosper under local environmental pressures. The aim of this study was to use RNA-Seq to perform genome-wide analysis of gene expression levels among known ecotypes of *E. vaginatum* originating from populations along a latitudinal gradient in a common garden. The primary goals are to (1) provide the first reference transcriptome available for the foundation arctic tundra species *E. vaginatum* during an extreme heat event and under normal summer temperature and (2) determine DEGs for ecotypes subject to an extreme heat event in relation to typical summer temperatures focusing primarily on HSPs and TFs.

## Results

### Transcriptome sequencing and de novo assembly

Sequencing generated 167,939,545 paired end reads, and after trimming for quality 120,794,728 reads remained across all samples. The complete set of reads were used to generate the *de novo* assembly that contained 423,353 transcripts with a combined total 323,059,790 assembled bases, 41.24% GC content, N50 of 1,441 bases and a median length of 373. Of the assembled transcripts, 97,236 (23%) mapped to probable contamination (e.g. fungus, bacteria) were removed. The 182,744 transcripts with significant hits mapped to plant species including *Ananas cosmo* (25,927, 14.2%), *Oryza sativa* (15,059, 8.2%), *Zea mays* (11,303, 6.2%) and *Elaeis guineensis* (10,999, 6.0%). The transcripts with significant matches to known proteins had a GC content of 40.9% and N50 of 2,070 bases. The remaining set of 143,373 transcripts with no significant BLAST results were combined with the transcripts with significant hits to form the final set of 326,177 unigenes for downstream analysis. These unigenes had a GC content of 40.9% and N50 of 1,601. The high-quality unigenes produced in this study have been deposited at: https://www.ncbi.nlm.nih.gov/sra/PRJNA555102

### Gene ontology (GO) classification

**Figure 1:**
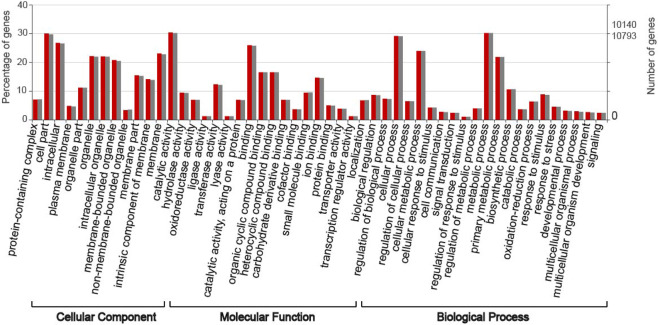
Gene Ontology (GO) classification for *Eriophorum vaginatum* unigenes with histogram representing overall percentage of unigenes found for GO terms in the three main functional categories. Red bars 26.6 °C day; grey bars 13.8 °C day.

Within the final unigene set, 124,150 were assigned GO terms resulting in a total of 286,156 GO terms recognised, including 93,296 (32.6%) assigned as biological function, 90,428 (31.6%) as cellular component, and 102,432 (35.8%) as molecular functions. The number of unigenes expressed in all categories were similar on both the 13.8 °C and 26.6 °C days. Figure 1 contains a bar graph with the percentage of unigenes for select GO terms represented at >1%. Notable GO terms to which unigenes were identified include: 2,325 that have transcription regulatory activity (GO:0140110), of which 1,961 had DNA-binding transcription factor activity (GO:0003700); 10,411 are identified as a response to stress (GO:0006950), of which 807 are a response to heat stress (GO:0009408); 71,427 are related to metabolic process (GO:0008152), of which 9,666 are related to regulation of metabolic processes (GO:0019222). All GO terms identified are in Supplemental Table S1.

### Differentially expressed genes

**Figure 2:**
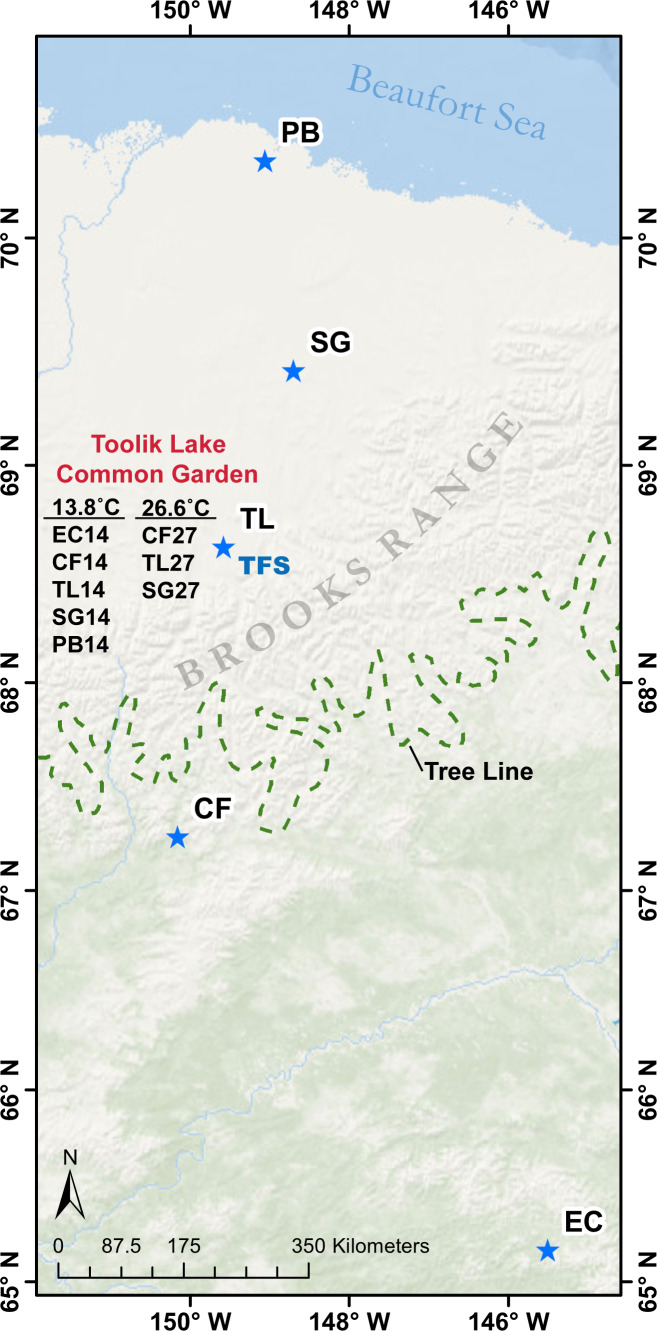
Map showing latitudinal origins of plant populations (EC, Eagle Creek; CF, Coldfoot; TL, Toolik Lake; SG, Sagwon; PB, Prudhoe Bay) in northern Alaska transferred to the Toolik Lake Common Garden. Five samples were taken from plants originating from these populations now in the common garden on a 13.8 °C day (EC14, CF14, TL14, SG14, PB14; July 18, 2016) and on a 26.6 °C day (CF24, TL24, SG24; July 13, 2016). Plants are found north and south of treeline (dashed green line). Toolik Field Station (TFS). Map created with ArcGIS Desktop 10.5.1. www.esri.com.

**Figure 3:**
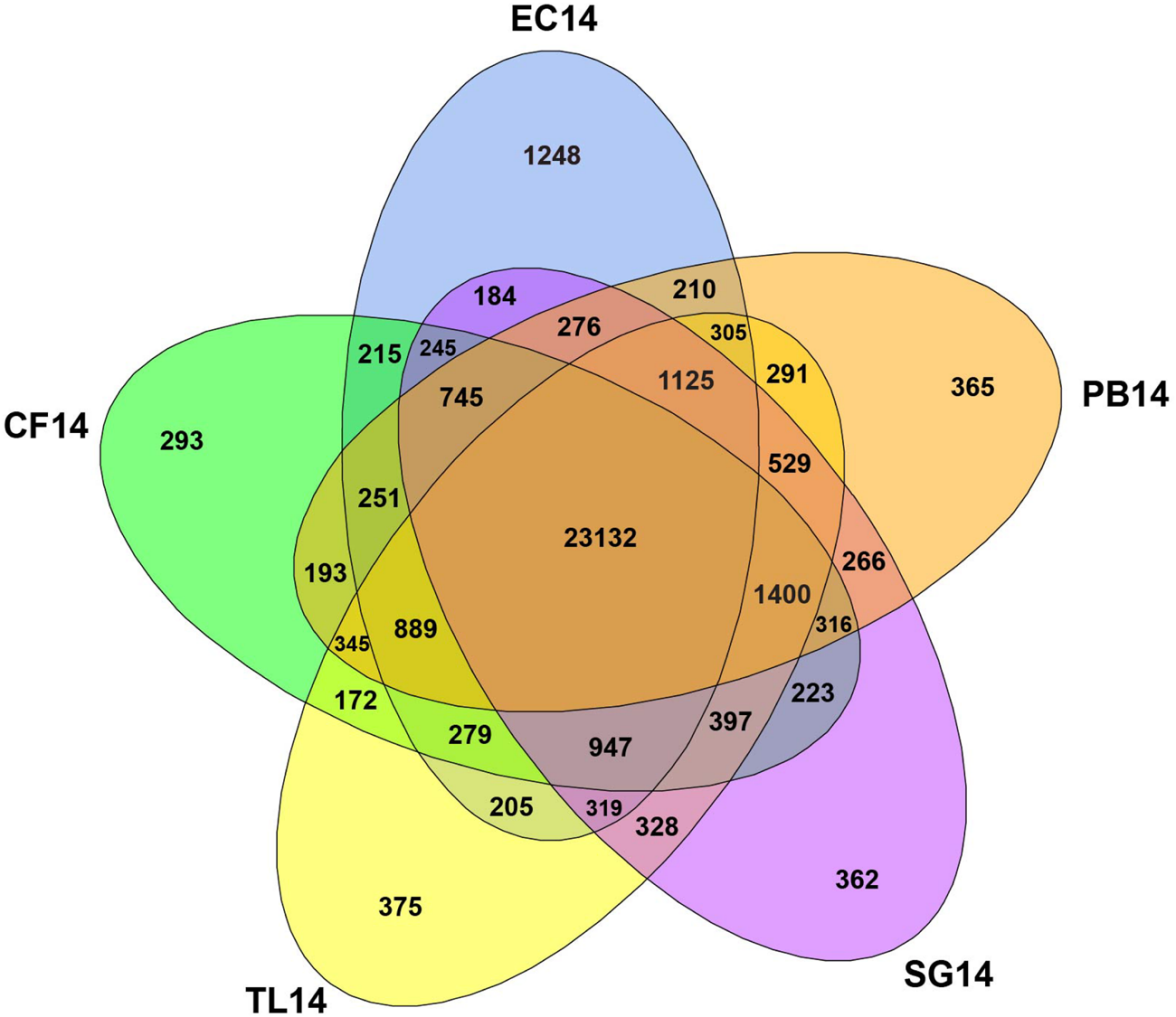
Venn diagram representing the number and overlap of unigenes expressed among five ecotypes of *Eriophorum vaginatum* on a 13.8 °C day in the TFS common garden. Eagle Creek (EC), Coldfoot (CF), Toolik Lake (TL), Sagwon (SG), Prudhoe Bay (PB).

After running *cuffdiff*, 36,999 unigenes had enough reads mapped to them to pass the initial statistical test within at least one sample for analysis of DEGs. These normalised unigenes were compiled into a matrix for further analysis. There were 36,439 unigenes found among the five ecotypes originating from populations along a latitudinal gradient in Alaska (Fig. 2; Eagle Creek [EC], Coldfoot [CF], Toolik Lake [TL], Sagwon [SG], Prudhoe Bay [PB], also see methods) sampled at the Toolik Lake Common Garden from the 13.8 °C day (hereafter referred to from south to north as EC14, CF14, TL14, SG14 and PB14) and of these 23,132 (63.5%) unigenes were expressed across all five samples. This amount increases to 28,247 (77.5%) unigenes expressed in at least four of the five samples. A total of 2,643 (7.3%) unigenes were found in only one of the five samples, ranging from 293 (0.8%) from CF14 to 1,248 (3.4%) from EC14 (>3× more than other ecotypes). There was comparable overall recovery among the five ecotypes sampled (EC14–30,575; CF14-30,042; TL14–31,038; SG14–30,794; PB14–30,273). See Fig. 3 for comparable overlap of unigenes expressed on the 13.8 °C day.

**Figure 4:**
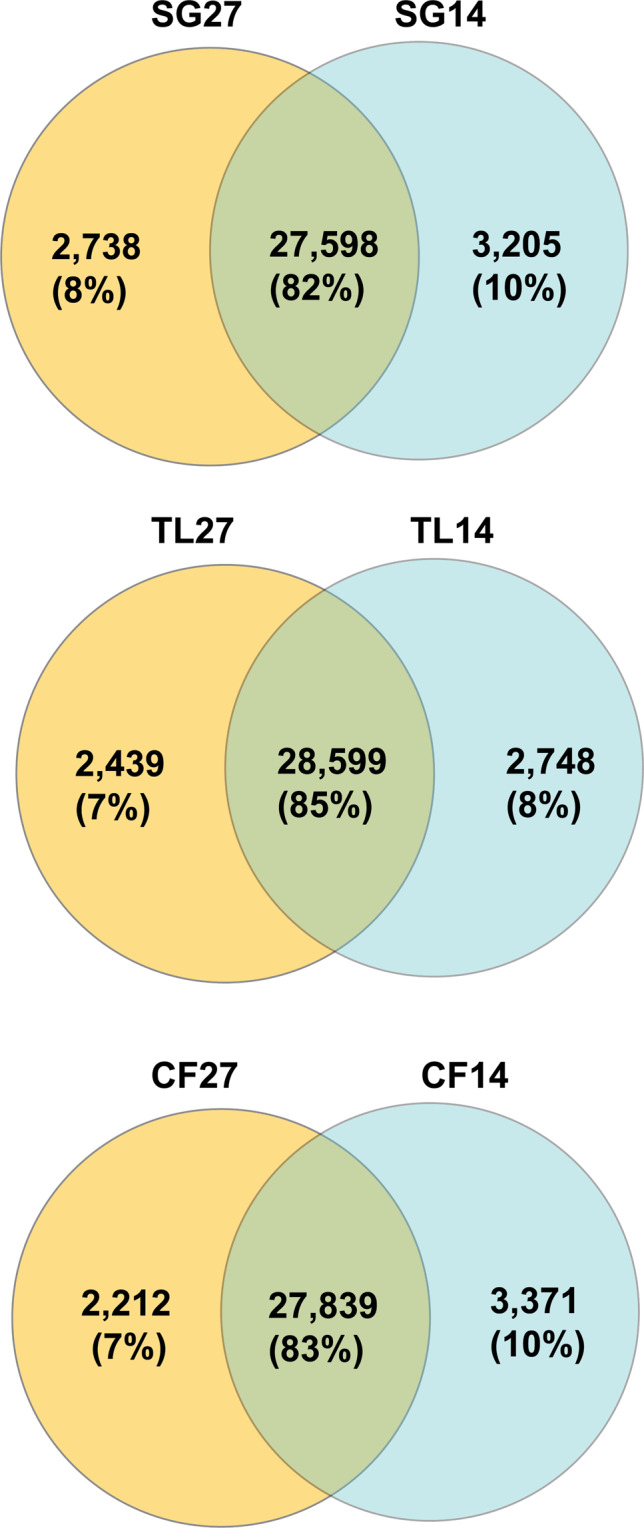
Venn diagram representing the number and overlap of unigenes expressed for ecotypes exposed to 13.8 °C vs. 26.6 °C days. Abbreviation as for Fig. 2 with numbers representing temperature day.

There were 33,422, 33,786 and 33,541 unigenes found for the CF, TL and SG ecotypes respectively from both sampling days combined (13.8 °C + 26.6 °C). Most unigenes were present both sampling days (82%-85%) with 8%-10% present only on the 13.8 °C day and 7%-8% present only on the 26.6 °C day (Fig. 4). There were 34,840 unigenes identified among the three ecotypes exposed to the 26.6 °C day (hereafter referred to from south to north as CF27, TL27, and SG27; Fig. 2), with 26,338 (75.6%) being expressed among all three ecotypes. There were 3,125 (9.0%) unigenes found in only one sample from the 26.6 °C day. TL27 shared more common unigenes with CF27 and SG27, 2,333 and 1,582 respectively, than the 1,462 unigenes CF27 and SG27 shared with each other.

**Figure 5:**
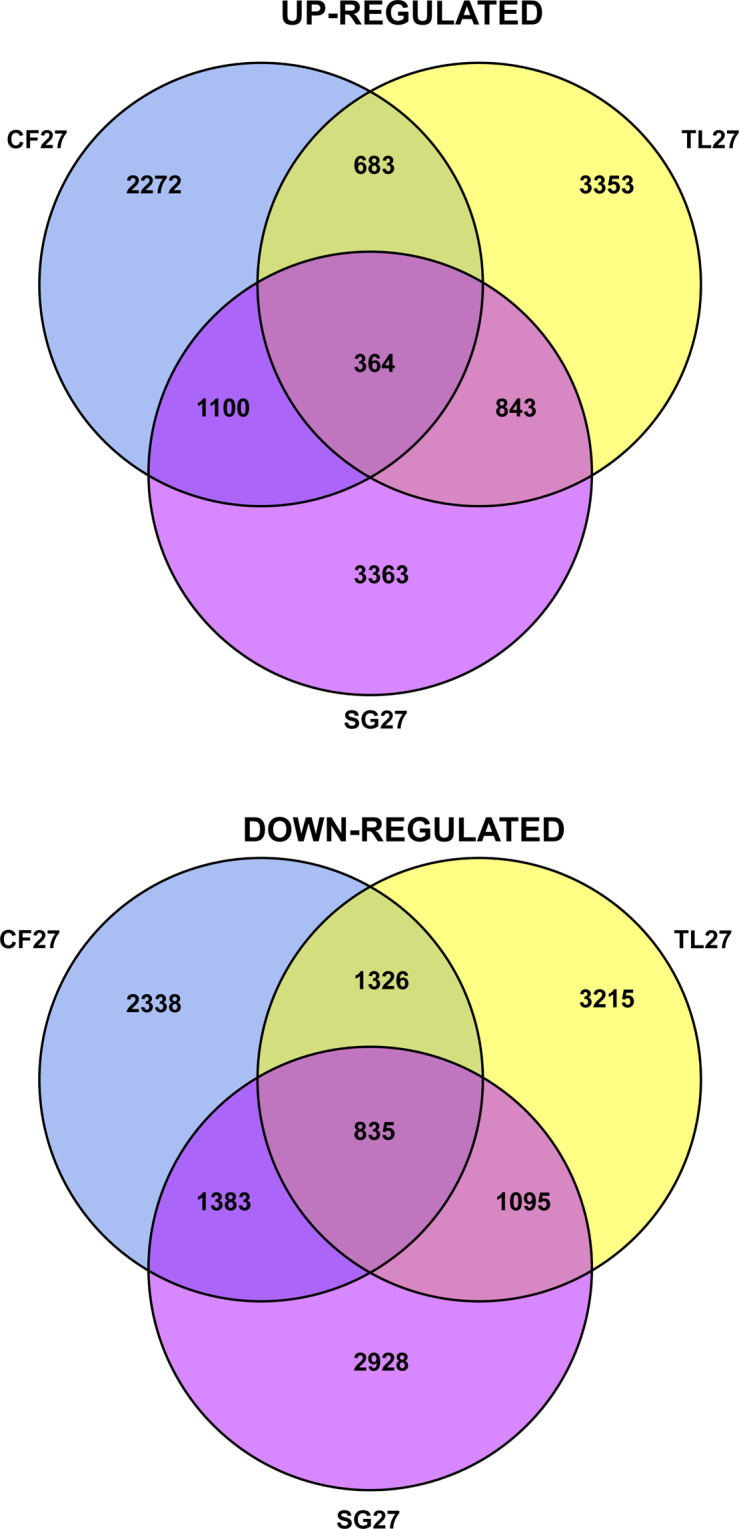
Venn diagram representing unigenes that are >2-fold up-regulated or down-regulated for *Eriophorum vaginatum* ecotypes on a 26.6 °C days compared to a 13.8 °C day. Abbreviation as for Fig. 2 with numbers representing temperature day.

Overall up-regulation and down-regulation was measured for the unigenes that were shared among the three ecotypes exposed to heat stress. Of those where there was>2-fold expression difference, 11,978 unigenes were up-regulated and 13,120 were down-regulated across ecotypes on the 26.6 °C day compared to the 13.8 °C day. A total of 364 unigenes were up-regulated and 835 down-regulated for all ecotypes. CF27 and SG27 shared more up-regulated and down-regulated unigenes than between TL27 and either CF27 or SG27 (Fig. 5).

**Figure 6:**
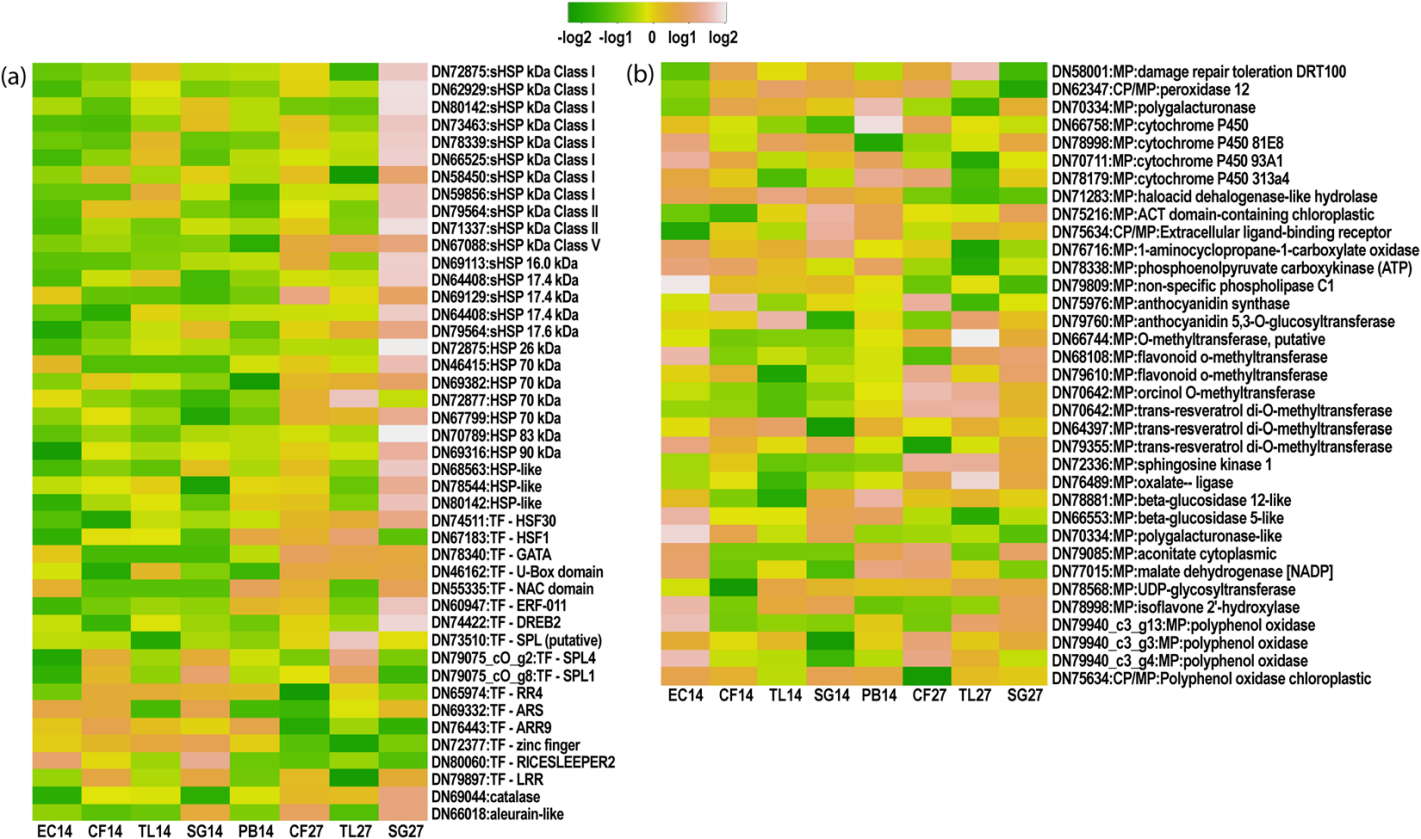
Heat maps representing unigene expression level for all ecotypes at both temperatures (13.8 °C and 26.6 °C) colour coded on a log scale for (**a**) Heat Shock Proteins (HSPs) and Transcription Factors (TFs) and (**b**) Selected Metabolic Processes (MP) and Cellular Processes (CP).

Individual normalised expression values were calculated across samples and those with >2-fold DEGs for HSPs, TFs, metabolic processes, and cellular processes were identified (Fig. 6, Supplemental Tables S2 and S3). Figure 6a illustrates the consistent up-regulation of large HSPs for all ecotypes on the 26.6 °C day. TL27 did not consistently up-regulate small HSPs (sHSPs), but sometimes these genes were down-regulated when up-regulated for both CF27 and SG27. TFs were either up-regulated or down-regulated consistently for ecotypes on the 26.6 °C day, but TL27 sometimes varied from the response of CF27 and SG27. TL ecotypes in general showed less DEGs between sampling temperatures than either CF or SG. There is >log2-fold variation among ecotypes for some important metabolic and cellular processes (Fig. 6b) with consistent DEGs between samples taken on the 13.8 °C and 26.6 °C day for these genes often affected during heat stress.

## Discussion

This study represents the first transcriptome for the foundation species of the moist acidic arctic tundra, tussock cottongrass, and utilises RNA-Seq to identify DEGs between ecotypes grown in a natural field-based common garden under typical and extreme heat conditions. The analyses revealed 36,439 unigenes with a range of 30,042 (CF14) to 31,038 (TL14). This is comparable to other such studies^41,42^. As expected most of the unigenes were identified to genomes of relatively closely related cultivated Monocot lineages of grasses (e.g. Rice, Corn; Supplemental Fig. 1). Most unigenes were expressed in all ecotypes (≈64%; Fig. 3), however several were discovered only for individual ecotypes, with the greatest proportion of these in EC14 (≈3%; >3× more than other ecotypes). EC originates from the southernmost latitude sampled and is isolated at a higher elevation (770 m) compared to other populations. Notable ecological or physiological differences from other southern populations have not been found for EC in common garden experiments^24,26^, but there are also potential cryptic differences from the other ecotypes through DEGs (Fig. 6; see below). This may be related to different environmental variables and the geographically isolated location of the EC home site compared to the more continuous distribution of *E. vaginatum* among the home sites of the other ecotypes. This is supported by population genetic analyses (Moody *et al*., unpublished data) showing that EC is genetically differentiated from other populations of *E. vaginatum* found along this Alaskan latitudinal gradient.

There was a relatively low percentage of unigenes recovered only on the different temperature days (Fig. 4) for the ecotypes exposed to heat stress. Variation in ecotype response between temperature days was evident through DEGs related to abiotic stress response, including HSPs and TFs (Fig. 6a; Supplemental Table 2). Here we focus primarily on HSPs and TFs as these can be directly related to abiotic stress. DEGs were also found for metabolic and cellular processes (Fig. 6b; Supplemental Table 2) on days with different temperatures as well as between different ecotypes, some of which have been related to heat stress response. It is important to point out that timing of leaf sampling can effects expression levels captured^11,43^. As we sampled at peak day temperature plant response of HSP and TFs were high and downstream metabolic responses may not have been active to a degree where DEGs was well defined. Timing can also be different among ecotypes^8^ and may to some extent explain differences found between the tundra ecotypes (TL and SG) during the extreme heat event (see below).

### Metabolic and cellular processes

The *E. vaginatum* ecotypes in the common gardens originate from populations in a tundra ecosystem north of treeline (TL, SG, PB) and a muskeg environment in taiga ecosystems south of treeline (EC, CF). Given the ecological variation observed from reciprocal transplant studies^24,29^, we expected that DEGs among ecotypes would show a strong correlation with the origin of the ecotype in taiga or tundra. While our focus is on HSPs and TFs we briefly discuss some pertinent DEGs with >log2-fold difference among ecotypes on both 13.8 °C and 26.6 °C days for genes involved in metabolic and cellular processes related to abiotic stress response. There was little or no correlation of DEGs with ecotypes originating from either taiga or tundra biomes. The southern-most and northern-most ecotypes (EC and PB, respectively) had comparably high expression levels for several genes (Fig. 6b). For example, EC14 and PB14 up-regulated cytochrome P450 and malate dehydrogenase (MDH) [NADP]-chloroplastic genes compared to the other ecotypes. The P450 genes are highly diverse and active in a wide range of metabolic processes including formation of primary and secondary metabolites^44^ and are known to be active stress response genes^45,46^. MDH is an important enzyme for cellular metabolism that has been shown to have upregulation under abiotic stress^47,48^. Given the difference in environments between the garden at TL and the EC and PB home sites, a stress-like response for these ecotypes may have affected regulation.

EC14 had pronounced up-regulation of several other metabolic and cellular process genes including non-specific phospholipase C1 (NPC1), aconitate-cytoplasmic, polygalacturonase-like, isoflavone 2’-hydroxylase (I2’H)-like, o-methyltransferases (OMTs) and polyphenol oxidase (PPO). Some of these are also up-regulated in heat-stressed plants. Phospholipase is important for growth and development as part of the phospholipid signaling network^49^. In *Arabidopsis* NPC1 has been recognised as important for abiotic stress resistance when up-regulated^49^. PPO catalyzes the reaction that leads to leaf browning and has been linked to abiotic stress reactions including leaf senescence. The direction of expression varies among studies. For example, up-regulation of PPO has been linked to increased vigor in rice^50^. However, both increase and decrease of PPO have been linked to drought stress^51^. Several of the genes up-regulated for EC14 are involved in the flavonol and isoflavonol pathways (cytochrome P450 93A, I2’H, and OMTs) and are up-regulated for plant defense^52^.

Several genes involved with metabolic processes were consistently down-regulated among the heat stressed ecotypes (CF27, TL27, SG27), namely, peroxidase 12, haloacid dehalogenase-like hydrolase, 1-aminocyclopropane-1-carboxylate oxidase, phosphoenolpyruvate carboxykinase (ATP), non-specific phospholipase C1 (NPC1) and polygalacturonase. Heat stress has been shown to lower peroxidase activity in cucumber^53^ and aminocyclopropane-1-carboxylate oxidase in wheat^54^ as part of an interaction that improves photosynthetic efficiency. NPC1 overexpression in *Arabidopsis* has been shown in plants with greater resistance to heat stress^49^, which goes counter to the heat stress response for these plants, in which NPC1 is down-regulated. However, most understanding of the NPC family of genes is limited to *Arabidopsis* and will benefit from additional targeted studies related to abiotic stress response^49^.

### Heat shock proteins and transcription factors

Under current climate change estimates the Arctic is warming faster than the rest of the planet with temperatures predicted to increase by as much as 11 °C by the end of the century^19^. It is important to identify how arctic plant ecotypes might react if exposed to higher temperatures, which have been infrequent in the past, but will have increased frequency in the coming decades^55^. The best studied genes with direct relation to heat stress response are HSPs and TFs, which are the primary focus for DEGs between the extreme heat event and typical summer temperature sampling. Among the ecotypes examined in the Toolik Field Station (TFS) common garden (Fig. 2), CF ecotypes originate from a native environment more frequently exposed to higher temperatures (>15 days/yr over 24 °C), whereas TL and SG ecotypes originate from environments that experience on average <3 days/yr of 24 °C or higher (some years it never reaches this temperature)^56,57^.

HSP expression is an indicator of plant response to abiotic stress, including heat and drought. If plants are adapted to heat stress, they should show up-regulation of HSPs in response to increased temperature^15,58,59^. HSPs are important as chaperones that stabilise structural proteins and repair damaged proteins. For crop plants that will be exposed to climate change, the discovery of varieties that will respond to heat stress by up-regulating HSPs has been an important line of research^8^. For example, some crop varieties have shown up-regulation of abiotic stress related genes under heat treatment and are considered to have better adaptation to heat stress^17,60,61^. The same may be expected for natural populations, as plants that evolve under environments exposed to more extreme temperature events would be expected to have a suitable response^17^.

As expected, we found up-regulation of HSPs on the 26.6 °C day across all ecotypes. Surprisingly, the plants in the common garden originating from the northernmost population (SG27) had comparably greater up-regulation of HSPs among the three ecotypes examined (Fig. 6a). There was also measured up-regulation of HSPs for CF27; expression levels were increased while remaining consistently lower than SG27. On the other hand, TL27 did not show consistent up-regulation of HSPs. Down-regulation of sHSPs was not uncommon, albeit at comparably low levels, whereas for larger HSPs (70–90 kDa) there was consistent up-regulation. Differences in HSP expression between TL14 and TL27 was low in comparison with the other ecotypes, which could indicate that the TL ecotype is not well adapted to respond to extreme heat events, which will be examined with future experimentation. Irrespective of the cause, these results do indicate that under heat stress these ecotypes are responding differently at the genomic level.

TFs are important in signaling pathways to alter plant functions that would adversely affect the plant under abiotic stress conditions^62^, and therefore are also indicators of stress response and often associated with HSP up-regulation. For this study we focused on TFs that have been recognised in heat stress response through DEGs in previous studies^17,63–65^ and had DEGs (>2-fold) under heat stress conditions for these ecotypes. TFs can be up-regulated or down-regulated under abiotic stress conditions in plants^10,42^ and in this study both occurred. Under heat stress, all ecotypes had up-regulation of heat shock factors (HSFs), GATA and U-Box, but for NAC, ethylene response factor (ERF), and dehydration response element binding (DREB) up-regulation was found for CF27 and SG27 only. Down-regulation was found for arginine-rich splicing (ARS), two component response regulator (ARR9), type-A response regulator 4 (RR4) across all ecotypes, but zinc finger proteins and SQUAMOSA promoter binding-like proteins (SPL4, SPL1) were down-regulated for CF27 and SG27 while SPL1 and SPL4 were upregulated only for TL27.

DREB, ERF and HSF have been shown to have correlative responses as part of pathways activated in response to multiple abiotic stresses^10,65^. DREB2 is upstream of HSFs and is important for regulation of their expression^63^. Overexpression of DREB2 in *Arabidopsis* has increased its tolerance to heat stress^66^. It was also found to be up-regulated and important in relation to other heat responsive genes in radish^65^, rice^62^, and wheat^8^. HSFs are up-regulated and interact with chaperone genes when heat stress is present^67^. Up-regulation of HSFs have been well documented for other plant lineages (e.g. radish^65^ and rice^68^). ERFs are part of a downstream response to ethylene, which is released by plants as a stress response and are associated with DREB up-regulation^69^. ERF11 is a core environmental stress response gene in *Arabidopsis*^70^ and was up-regulated by *E. vaginatum* ecotypes here. NAC genes are also up-regulated in relation to DREB in response to drought stress in *Arabidopsis*^71^, multiple abiotic stresses in crops (e.g. rice and wheat^72^) and among Douglas-fir ecotypes^73^.

Among other up-regulated TFs GATA genes are part of a conserved family found across organisms. A recent study in rice found multiple GATA variants responsive to abiotic stresses with some individual variants able to respond to multiple stresses^74^; GATA26, up-regulated here, was found to be responsive to osmotic and drought stress. U-Box genes are also upregulated in response to abiotic stress^75^, and in combination with UDP-glucosyltransferase have shown a correlated response considered important for improving thermotolerance of economically important crop plants^63^. The correlated up-regulation of these genes suggest they could have an important role for natural populations as well.

Another abiotic-stress related gene that was up-regulated across ecotypes includes the regulatory gene catalase (CAT), which is an antioxidant enzyme that acts under heat stress by reducing O_2_ and H_2_O_2_ accumulation in plants^62,65^. It has been considered necessary for detoxification of reactive oxygen species (ROS) during abiotic stress^76^ and has been found to play a role in major grain crops^61,77^. Likewise, aleurain was up-regulated in CF27 and SG27 and has been associated with drought stress and leaf senescence^78–80^.

Among the several TFs down-regulated for the ecotypes under heat stress, zinc finger are a family of regulatory proteins involved in drought stress reactions in plants^81^ and can help regulate ABA^82,83^. For *E. vaginatum*, we found down-regulation of two identified zinc finger proteins under heat stress in SG27 and CF27. One of these was identified as a RICESLEEPER 2-like gene. The RICESLEEPER genes are part of a family of transposases that include a NAC domain and heat shock cognates (HSCs) that are part of an abiotic stress response in *Amaranthus*^84^ and has been associated with ABA interaction in sugarcane^85^.

Other TFs down-regulated during heat stress are less well known in relation to abiotic stress. Leucine-rich repeat receptors (LRR) can interact with HSP90 under abiotic stress^86^. It was down-regulated for the *E*. *vaginatum* ecotypes, corresponding with up-regulation of HSP90. Likewise, response regulators (ARR9, RR4) were down-regulated in all ecotypes. RR4 is a probable cytokinin response gene^87^ which in turn is important for heat stress response in plants^88^. ARR9 has not previously been reported as a heat stress response gene, but ARR family genes were recognised as differentially expressed in Korean Fir under heat stress treatments^42^.

SPL family genes can part of a miRNA pathway for heat shock memory, that can be downregulated in response to heat stress^43,89^. Both SPL4 and SPL1 were upregulated in TL27, while down-regulated in CF27 and SG27, however other TFs in the pathway such as Argonaute^43^ did not show DEGs. Up-regulation of SPL4 has been associated with suppression of reproductive budding and vegetative tillering in *Panicum*^90^, while down-regulation was associated with reproductive bud initiation, but not specifically associated to heat stress. Tillering is the primary form of new growth for *E. vaginatum* and further exploration of genes related to abiotic stress in relation to tiller production will be an important avenue of exploration in future work. SPL1 has been associated with thermotoleration in *Arabidopsis* related to reproductive buds^91^. Reproductive process was not a focus of this work, as only leaf material was sampled, but will be of interest in future work.

These results confirm that all ecotypes responded to the extreme heat event through differential expression of HSPs and TFs. However, there was variation among ecotypes. It is striking that SG27 had a much stronger response than the other ecotypes, although it originated from the northern-most part of the range, where >24 °C is rare. While it may be beneficial that this ecotype responds to heat stress, there are energetic costs. It is plausible that if the SG ecotype is exposed more regularly to higher temperatures, as opposed to a rare event, reduced fitness over time could be an outcome. The other tundra ecotype (TL) had a relatively weak response to heat stress indicating a potential lack of adaptation for increases in temperature in the Arctic over the coming decades. However, the TFS common garden is the home site for TL and the abiotic stress response for this ecotype could reflect home site adaptations in gene regulation and plant/environment interactions that are currently unknown. For example, the TL ecotype may have different timing of expression in relation to heat stress and the timing of sampling may not have captured the peak gene expression levels. The intermediate response of CF plants, that more frequently see higher temperatures, may exemplify a well-adapted response to heat stress. This is an important first step in gaining an understanding of genomic level response of *E. vaginatum* ecotypes and assessing their performance under climate change.

## Ecological Consequences

From an ecological perspective, there is concern that regionally adapted ecotypes will be at a disadvantage in their current range, especially in the arctic tundra where the effects of climate change are extreme and parts of the Arctic are seeing dramatically higher summer temperatures. Long term studies using reciprocal transplant gardens show there is greater survival of *E. vaginatum* in the Arctic by plants at their home site and by southern ecotypes that are moved north^24^. However, studies that use traditional ecological measures provide limited evidence for advantage for plants from southern populations moved north as well as sometimes contrasting results for home site advantage^29,92,93^. The results of this study confirm consistent differential response to heat stress among ecotypes originating from different points along a latitudinal gradient that spans tundra and taiga ecosystems. This only occurred when plants were exposed to extreme temperatures, but not to average summer temperatures, which could signify some are better adapted than others as temperatures increase in the Arctic.

Ecotype variability will be particularly important for predictive accuracy of vegetation distribution models under climate change. While most models don’t use ecotype specific information to predict distributions in relation to climate change, the ability to use genetically informed environmental niche models can improve accuracy^94,95^. This will be particularly important for taxa that are considered foundation species in ecosystems, such as *E. vaginatum*, and which can be widespread and highly variable across their ranges^22,24,92^. Understanding how these ecotypes are adapted to specific niches and how this relates to physiological response down to the molecular level can provide important information relevant to carbon cycles and nutrient storage across the Arctic^92^. Therefore, it is important to consider cryptic variation that may be observed through DEGs under both common garden and growth chamber studies.

As extreme weather events increase across the globe, studies that capture plant response to extreme abiotic events will provide insight into how a species and its ecotypes will respond. This research has uncovered differences between ecotypes in potential adaptation to heat stress and should be followed by a combination of reciprocal garden and growth chamber experiments that control for multiple time intervals and temperatures as well as undertaking simultaneous measurement of physiological responses to assess ecotype responses under specific environmental changes. Comparisons between field studies and controlled growth chamber studies should provide corroboration of the differences among the ecotypes, but also help to estimate the environmental effect. Homesite advantage has been shown for all the ecotypes examined here in long-term ecological studies and may have a role in differential response found for *E. vaginatum*. In combination with more traditional measures, genomic tools can help to determine the role of local adaptation and the response of ecotypes to climate change more precisely.

## Methods

### Sampling

*Eriophorum vaginatum* from six populations along a latitudinal gradient of ≈1° intervals (65.43°N-70.33°N) in northern Alaska were transplanted into adjacent common gardens^29^ at the remote Arctic Toolik Field Station (TFS; 68.63°N, 149.58°W, Fig. 2) in 2012 and 2013. Mature plants form tussocks of approximately 300–600 tillers^96^ with new tillers producing 1–4 leaves per season^97^. All new leaves from individual tillers were collected from the common gardens during peak day temperatures (between 2–3 pm) from three accessions each from five latitudinal ecotypes on a 13.8 °C ambient day (7/16/16; EC14 [65.43°N, 145.52°W]; CF14 [67.26°N, 150.17°W]; TL14; SG14 [69.42°N, 148.70°W]; PB14 [70.33°N, 149.06°W]) and from 3 accessions each from 3 ecotypes on a 26.6 °C ambient day (7/13/16; CF27; TL27; SG27). The latter is an extreme temperature for the Alaskan Arctic at the natural home sites for the plants (average mean July temperature 2012–2016: CF = 14.2 °C, TL = 10.8 °C, SG = 10.6 °C)^93^. Ambient temperature >24 °C is more commonly reached at CF during the summer, while it is rare for TL and SG (Average number days/year >24 °C June-August 2010–2017: CF = 15.6, TL = 1.3, SG = 2.6). The high temperature on the day of sampling (26.6 °C) was the 2^nd^ highest temperature recorded at TFS over the previous 10 years^57^. Previous studies have recognized an ecotype north of treeline (PB, TL, SG) and south of treeline (CF, EC) that have different ecological responses in long-term reciprocal transplant garden studies while each population also retains homesite advantage regarding long-term survival and flowering^24,25,27,29,92,93^. After whole leaves were collected fresh from the common garden, they were immediately immersed into falcon tubes filled with liquid nitrogen. Leaf sampling was limited as to minimize damaging plants at the common garden involved in long-term ecological studies. After samples were flash frozen, the tubes were placed in coolers containing dry ice and transferred to -80 °C freezers at TFS. The samples were transferred via overnight mail in dry ice to the University of Texas El Paso (UTEP) where they were stored in -80 °C freezers until RNA extractions were performed.

### Library preparation and RNA-sequencing

RNA was extracted from 300 mg leaf material using the RNeasy Plant Mini Kit (QIAGEN). Most extractions required all field-sampled material to attain quantities for transcriptome sequencing. RNAs from each accession were pooled in equal quantities to represent the sampled populations. All stages of library preparation were performed at the Genome Sequencing and Analysis Facility (GSAF) at the University of Texas (Austin, TX). Total RNA was processed using the Ribo-Zero rRNA removal kit (Epicentre). Final library quality was accessed using the Agilent BioAnalyzer DNA 7500 chip and qPCR using Kapa’s SYBR Green kit for Illumina libraries. The samples were sequenced using the Illumina HiSeq. 4000 platform on a Paired-End run (PE150) at GSAF.

### De novo assembly and functional annotation

Raw sequences were quality checked using FastQC (www.bioinformatics.babraham.ac.uk/projects/fastqc/). Sequences were then used as input into the program Trinity to create a *de novo* transcriptome of the collected leaf samples^98^. Functional annotation of the assembled unigenes was performed using Blast2GO^99^; using default parameters. In short, for each unigene a BLASTX search against the NCBI non-redundant database (Nr) was performed. Sequences with significant hits were then mapped to a Gene Ontology (GO) annotation database within Blast2GO. An InterProScan search was performed using EMBL-EBI InterPro^100^ within the Blast2GO program to supplement the Blast findings for the functional annotation. Venn diagrams describing the overlap of unigenes found in each sample were constructed in R (version 3.5.2)^101^ using the package *VennDiagram*^102^.

### Analysis of differentially expressed genes

Clean reads were mapped to the constructed reference transcriptome using Bowtie2^103^. Expression levels were normalised within Cufflinks^104^. The comparison of the sites for detection of differential expression was completed using the Cufflink’s *cuffdiff* command using the default parameters that generated the Fragments Per Kilobase of unigenes per Million fragments mapped (FPKM) values. For the comparisons, unigenes were considered significant when there was greater than 2-fold change in the FPKM values. FPKM were extracted from the Cuffdiff output file using in-house scripts for use in specific heatmap construction using the R package *heatmap3*^105^. Heat maps display genes with at least log2-fold change. Relevant GO terms containing at least 1% of the unigenes found on either the 13.8 °C or 26.6 °C day were plotted using WEGO^106^. In-house python scripts were used to isolate genes associated with various functional attributes for further data mining.

## Supplementary information

Supplementary Information.

Supplementary Information 2.
