# Supplementary material for: Comparative transcriptomics of an arctic foundation species, tussock cottongrass (Eriophorum vaginatum), during an extreme heat event
Source: Sci Rep. 2020 Jun 2;10:8990. doi: 10.1038/s41598-020-65693-8 (PMC7265556; doi:10.1038/s41598-020-65693-8)
Supplement: Supplementary file 1 — Supplementary Information. [file 41598_2020_65693_MOESM1_ESM.pdf]

Supplementary Information

Article in *Scientific Reports*

Comparative transcriptomics of an arctic foundation species, tussock cottongrass (*Eriophorum vaginatum*), during an extreme heat event

Jonathon E. Mohl[1], Ned Fetcher[2], Elizabeth Stunz[3], Jianwu Tang[4], Michael L. Moody[3*]

[1]Bioinformatics Program, University of Texas at El Paso, El Paso, TX 79968, USA
[2]Institute for Environmental Science and Sustainability, Wilkes University, Wilkes-Barre, PA 18766, USA
[3]Biological Sciences, University of Texas at El Paso, El Paso, TX 79968, USA
[4]The Ecosystems Center, Marine Biological Laboratory, Woods Hole, MA 02543, USA
***Correspondence Author**: (mlmoody@utep.edu)

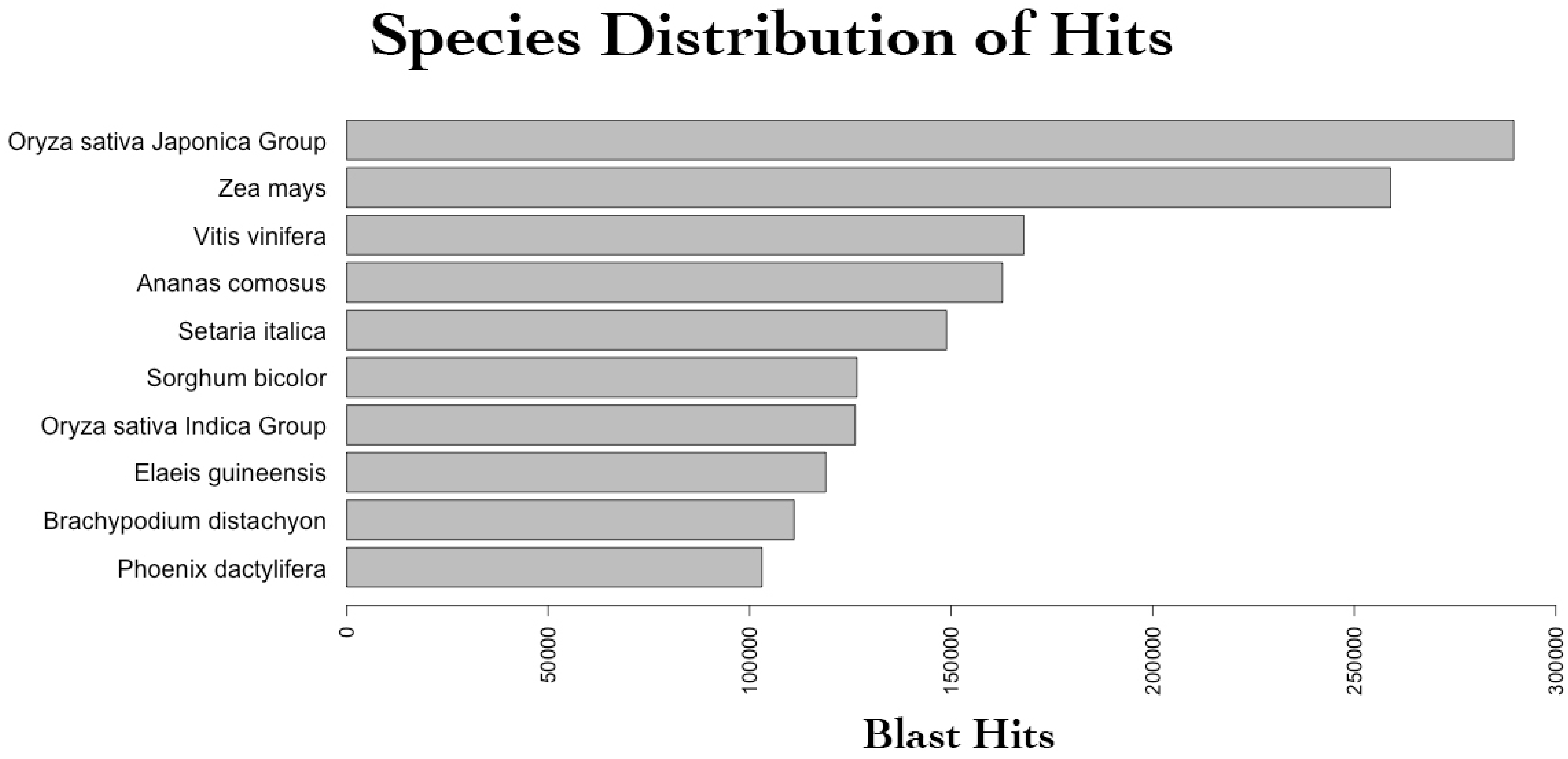

Supplemental Figure S1. Bar graph representing the ten most common species for which Gene Ontology matches were found.

Supplemental Table S1. Relative gene expression for HSPs and TFs for *Eriophorum vaginaum* ecotypes on a 13.8°C (EC14, CF14, TL14, SG14, PB14) and 26.6°C (CF27, TL27, SG27) day at the Toolik Field Station common-garden.

Contig	Gene Description	EC14	CF14	TL14	SG14	PB14	CF27	TL27	SG27
TRINITY_DN72875_c1_g5_i4	small heat shock protein	3	6	38	10	11	24	1	636
TRINITY_DN62929_c0_g2_i2	small heat shock protein, chloroplastic-like isoform X2	9	22	37	17	19	46	27	433
TRINITY_DN80142_c24_g3_i2	kDa class I heat shock -like	294	143	369	639	324	180	164	4336
TRINITY_DN73463_c1_g1_i1	kDa class I heat shock -like	35	31	98	471	154	437	102	6069
TRINITY_DN78339_c1_g5_i1	kDa class I heat shock -like	84	70	442	79	92	325	183	2684
TRINITY_DN66525_c0_g3_i1	kDa class I heat shock -like	14	29	81	18	41	49	41	438
TRINITY_DN59856_c0_g2_i1	kDa class I heat shock	15	16	166	49	8	50	47	973
TRINITY_DN79564_c0_g1_i1	kDa class II heat shock -like	0	20	21	2	0	10	2	403
TRINITY_DN71337_c0_g1_i1	kDa class II heat shock -like	1	8	3	10	7	20	4	519
TRINITY_DN67088_c0_g7_i2	kDa class V heat shock	3	5	3	3	1	30	45	81
TRINITY_DN69113_c0_g4_i4	16.0 kDa heat shock protein, peroxisomal	5	6	8	10	11	25	8	80
TRINITY_DN64408_c0_g1_i2	17.4 kDa class III heat shock protein	2	6	11	2	4	6	6	72
TRINITY_DN69129_c0_g1_i1	DNAJ heat shock N-terminal domain-containing protein	75	35	34	29	39	149	67	128
TRINITY_DN64408_c0_g1_i1	17.4 kDa class III heat shock protein	20	10	57	38	41	42	43	313
TRINITY_DN79564_c0_g12_i1	class II small heat shock protein Le-HSP17.6	0	7	29	103	6	58	148	805
TRINITY_DN72875_c1_g5_i9	heat shock HSP26	133	294	506	405	302	420	408	8601
TRINITY_DN46415_c0_g1_i1	heat shock protein 70	8	0	0	0	4	6	4	46
TRINITY_DN69382_c0_g5_i1	heat shock protein 70	50	163	93	51	7	208	246	493
TRINITY_DN72877_c2_g6_i1	Heat shock cognate 70 kDa protein 1	196	131	108	78	129	266	591	165
TRINITY_DN67799_c0_g1_i1	heat shock 70 kDa protein, mitochondrial-like	38	56	40	18	33	77	73	138
TRINITY_DN70789_c0_g2_i5	heat shock 83	13	26	16	32	35	42	30	862
TRINITY_DN69316_c1_g1_i2	heat shock protein 90	1	27	16	26	19	41	24	417
TRINITY_DN68563_c2_g1_i2	kDa heat shock -like	3	7	4	41	12	44	8	682
TRINITY_DN74511_c0_g1_i5	heat shock factor HSF30-like	4	2	8	7	8	14	16	32
TRINITY_DN78340_c3_g7_i1	GATA transcription factor 26-like	10	0	0	0	4	31	21	19
TRINITY_DN46162_c0_g3_i1	U-box domain-containing protein 33-like	38	10	61	24	13	76	72	79
TRINITY_DN55335_c0_g4_i1	NAC domain-containing 83-like	34	0	0	0	91	34	0	84
TRINITY_DN60947_c0_g1_i1	ethylene-responsive transcription factor ERF011-like	11	15	18	17	33	31	15	75
TRINITY_DN74422_c0_g5_i1	DREB protein 2 isoform c	92	42	99	76	60	112	78	363
TRINITY_DN65974_c0_g1_i3	RR4 - Corn type-A response regulator	25	61	59	56	54	11	44	29
TRINITY_DN69332_c0_g3_i4	Serine/arginine-rich splicing factor RS2Z33	127	175	132	117	170	43	85	48
TRINITY_DN76443_c0_g6_i3	two-component response regulator ARR9-like	69	84	101	119	68	27	14	34

TRINITY_DN72377_c0_g2_i1	zinc finger protein	261	193	3	308	3	2	42	118
TRINITY_DN80060_c3_g1_i8	zinc finger BED domain-containing RICESLEEPER 2-like	201	63	35	280	23	20	33	17
TRINITY_DN79897_c1_g12_i2	putative leucine-rich repeat receptor-like protein kinase -like	28	74	31	72	22	56	8	66
TRINITY_DN69044_c0_g3_i1	catalase	241	1175	1115	246	1106	1844	1746	4364
TRINITY_DN66018_c0_g1_i2	aleurain-like	39	10	16	1722	26	5224	8	11500

Supplemental Table S2. Relative gene expression with >2× DIGs among *Eriophorum vaginaum* ecotypes on a 13.8°C (EC14, CF14, TL14, SG14, PB14) and 26.6°C (CF27, TL27, SG27) day at the Toolik Field Station common-garden.

Contig	Gene Description	EC14	CF14	TL14	SG14	PB14	CF27	TL27	SG27
TRINITY_DN42318_c1_g3_i1	probable LRR receptor-like serine/threonine-protein kinase	138	118	43	172	145	22	19	61
TRINITY_DN42448_c0_g3_iS1	putative wall-associated receptor kinase-like 16	28	52	0	17	7	53	18	7
TRINITY_DN44244_c0_g2_i1	Wall-associated receptor kinase 5	9	43	22	22	38	14	20	32
TRINITY_DN44888_c0_g1_i3	probable LRR receptor-like serine threonine- kinase	224	8	0	44	0	0	0	0
TRINITY_DN46162_c0_g1_i1	U-box domain-containing protein 33-like	44	78	14	48	59	18	86	45
TRINITY_DN46162_c0_g3_i1	U-box domain-containing protein 33-like	38	10	61	24	14	76	72	79
TRINITY_DN46415_c0_g1_i1	heat shock protein 70	8	0	0	0	4	6	4	46
TRINITY_DN46632_c0_g2_i1	Wall-associated receptor kinase 3	3	0	0	0	17	0	0	5
TRINITY_DN49013_c0_g1_i1	Wall-associated receptor kinase 3	23	33	0	48	0	35	7	0
TRINITY_DN50617_c0_g1_i1	Wall-associated receptor kinase 3	13	11	16	6	4	6	12	5
TRINITY_DN50820_c0_g1_i1	ethylene-responsive element binding factor	10	0	0	0	0	174	0	0
TRINITY_DN50943_c0_g1_i1	cysteine-rich receptor-like protein kinase 6	54	35	36	7	50	127	18	72
TRINITY_DN51605_c0_g2_i1	OAY78020.1Receptor-like serine/threonine-protein kinase SD1-8	14	11	8	12	13	3	9	6
TRINITY_DN51605_c0_g3_i1	OAY78020.1Receptor-like serine/threonine-protein kinase SD1-8	19	11	8	2	14	0	6	0
TRINITY_DN51965_c0_g1_i2	peroxidase 52	0	2	0	0	0	4	8	0
TRINITY_DN52018_c0_g1_i1	Lectin-domain containing receptor kinase A4.2	12	2	7	5	18	9	5	0
TRINITY_DN52808_c0_g1_i1	MDIS1-interacting receptor like kinase 2-like	241	31	55	66	0	0	25	60
TRINITY_DN54313_c0_g1_i1	LRR receptor-like serine/threonine-protein kinase EFR	0	126	10	0	0	8	28	11
TRINITY_DN54401_c0_g1_i1	Disease resistance family protein / LRR family protein	61	5	4	18	16	13	11	9
TRINITY_DN54517_c0_g2_i1	retrotransposon unclassified	0	0	0	0	0	50	0	0
TRINITY_DN54602_c0_g6_i1	disease resistance	3	0	43	0	0	0	0	0
TRINITY_DN55283_c0_g2_i1	disease resistance	0	0	0	49	35	149	130	13

TRINITY_DN55335_c0_g4_i1	NAC domain-containing 83-like	34	0	0	0	91	34	0	84
TRINITY_DN55807_c0_g2_i1	rust resistance kinase Lr10-like isoform X1	40	59	13	32	116	41	0	0
TRINITY_DN57120_c0_g2_i1	predicted protein	58	19	13	18	42	54	23	14
TRINITY_DN57120_c0_g3_i1	predicted protein	76	11	5	0	0	8	9	5
TRINITY_DN57716_c0_g1_i1	predicted protein	0	16	9	8	3	13	38	1
TRINITY_DN57762_c0_g2_i1	predicted protein	133	30	55	63	127	91	56	37
TRINITY_DN58450_c0_g3_i1	kDa class I heat shock -like	68	1256	105	529	140	771	0	7058
TRINITY_DN58683_c0_g1_i1	peroxidase	26	113	238	14	91	119	54	36
TRINITY_DN58683_c0_g2_i1	JAT56219.1Peroxidase 12	10	89	142	33	39	63	18	0
TRINITY_DN58882_c0_g1_i1	LRR receptor-like serine threonine- kinase GSO2	31	171	0	0	0	0	0	0
TRINITY_DN59100_c0_g2_i1	probable LRR receptor-like serine threonine- kinase	42	0	0	0	110	105	0	67
TRINITY_DN59171_c0_g4_i1	ATP synthase subunit alpha, mitochondrial isoform X2	5	0	0	0	0	0	0	2
TRINITY_DN59191_c0_g1_i1	AGH18692.1shikimate kinase-like protein	0	5	8	7	9	11	13	7
TRINITY_DN59310_c1_g4_i1	probable LRR receptor-like serine/threonine-protein kinase	20	44	160	0	59	47	73	261
TRINITY_DN59362_c0_g1_i2	NAD kinase 1	7	8	16	2	5	4	5	3
TRINITY_DN59393_c1_g1_i1	catalase	21	0	0	0	0	0	0	0
TRINITY_DN59748_c0_g3_i1	putative LRR receptor-like serine/threonine-protein kinase	38	26	20	14	16	36	27	25
TRINITY_DN59856_c0_g2_i1	kDa class I heat shock	15	16	166	49	8	50	48	973
TRINITY_DN59864_c0_g1_i1	predicted protein	11	3	3	9	10	6	7	27
TRINITY_DN59999_c0_g3_i3	probable leucine-rich repeat receptor kinase	17	0	4	0	32	20	0	13
TRINITY_DN60196_c0_g3_i3	predicted protein	6	8	9	5	2	5	0	2
TRINITY_DN60196_c0_g3_i4	PREDICTED: uncharacterized protein LOC109504969	0	9	5	12	15	20	11	1
TRINITY_DN60286_c0_g1_i1	Cysteine-rich receptor-like protein kinase 10	25	34	35	0	0	38	66	33
TRINITY_DN60688_c0_g2_i1	Zinc-binding alcohol dehydrogenase domain-containing protein 2	4	66	11	6	18	21	14	0
TRINITY_DN60793_c0_g2_i1	NBS domain resistance	0	0	0	0	0	165	0	0
TRINITY_DN60918_c0_g1_i1	predicted protein	42	17	56	17	64	36	14	16
TRINITY_DN60947_c0_g1_i1	ethylene-responsive transcription factor ERF011-like	11	15	18	17	33	31	16	75
TRINITY_DN61000_c0_g6_i1	probable receptor-like protein kinase	80	30	7	6	7	11	6	21
TRINITY_DN61082_c0_g1_i1	wall-associated receptor kinase 5-like	11	12	152	0	42	17	49	23
TRINITY_DN61085_c0_g1_i1	peroxidase P7-like	0	5	4	12	1	10	32	1
TRINITY_DN61312_c0_g3_i1	receptor kinase-like protein Xa21	41	92	145	0	264	113	38	90
TRINITY_DN61405_c0_g1_i1	putative NBS-LRR protein	0	0	0	0	0	32	0	17
TRINITY_DN61432_c0_g1_i1	Chitin elicitor receptor kinase 1	0	7	9	5	12	7	27	25

TRINITY_DN61432_c0_g3_i1	Chitin elicitor receptor kinase 1	14	10	0	38	4	32	12	14
TRINITY_DN61432_c0_g6_i1	Chitin elicitor receptor kinase 1	22	5	44	26	25	24	20	37
TRINITY_DN61980_c0_g5_i1	TPA: putative lectin-like receptor protein kinase family protein	17	13	14	7	6	4	4	5
TRINITY_DN61984_c0_g8_i1	Receptor-like serine threonine- kinase SD1-8	36	24	64	0	28	0	0	145
TRINITY_DN61994_c0_g1_i1	calmodulin 3b (phosphorylase kinase, delta)	10	14	33	16	21	19	15	3
TRINITY_DN62156_c0_g2_i1	OAY83756.1Receptor-like protein 12	119	12	0	76	205	4	5	56
TRINITY_DN62156_c0_g5_i1	Receptor 12	82	0	132	0	0	0	0	17
TRINITY_DN62340_c0_g1_i3	probable serine/threonine-protein kinase	16	29	21	23	16	26	28	16
TRINITY_DN62364_c0_g1_i1	kinase superfamily protein	3	3	1	1	3	4	2	1
TRINITY_DN62602_c0_g5_i1	Disease resistance (CC-NBS-LRR class) family	30	0	0	468	31	0	0	0
TRINITY_DN62702_c0_g10_i1	probable LRR receptor-like serine/threonine-protein kinase	69	15	14	17	0	0	0	14
TRINITY_DN62702_c0_g1_i1	probable LRR receptor-like serine/threonine-protein kinase	131	141	31	163	77	10	28	104
TRINITY_DN62702_c0_g6_i1	predicted protein	0	0	4	31	10	14	0	0
TRINITY_DN62702_c0_g9_i1	predicted protein	21	49	38	0	53	8	22	33
TRINITY_DN62833_c0_g1_i1	putative kinase-like protein TMKL1	3	12	7	6	3	7	17	5
TRINITY_DN62902_c0_g1_i1	receptor kinase 1	3	1	3	2	1	2	3	2
TRINITY_DN62929_c0_g2_i2	small heat shock protein, chloroplastic-like isoform X2	9	22	37	17	19	46	27	433
TRINITY_DN63053_c0_g1_i1	probable leucine-rich repeat receptor kinase At1g68400	1	12	8	10	1	7	32	1
TRINITY_DN63165_c0_g5_i1	predicted protein	62	31	23	55	23	0	13	30
TRINITY_DN63165_c0_g6_i1	predicted protein	89	12	0	0	0	26	0	119
TRINITY_DN63165_c0_g8_i1	predicted protein	4	0	42	18	0	0	0	22
TRINITY_DN63252_c1_g4_i1	cationic peroxidase SPC4-like	26	32	12	53	16	90	4	39
TRINITY_DN63252_c1_g5_i1	XP_010912023.1 peroxidase 12-like	0	4358	10095	1481	1870	16124	3454	4080
TRINITY_DN63252_c1_g6_i1	cationic peroxidase SPC4-like	0	41	5	9	133	33	19	28
TRINITY_DN63482_c0_g1_i3	transcription factor ILI3-like	0	0	0	0	1	3	11	0
TRINITY_DN63575_c0_g6_i1	receptor 12	0	155	4	400	52	19	23	166
TRINITY_DN63674_c0_g1_i2	transcription factor TT2-like	0	0	0	0	0	5	36	0
TRINITY_DN63825_c0_g1_i1	aconitate hydratase, cytoplasmic-like	3	1	2	2	1	1	2	10
TRINITY_DN63991_c0_g1_i3	inositol 3-kinase-like	3	2	2	2	2	4	4	1
TRINITY_DN64338_c0_g4_i1	probable LRR receptor-like serine threonine- kinase	0	0	66	45	0	19	5	6
TRINITY_DN64361_c0_g1_i2	peroxidase 64-like	6	6	12	16	4	6	27	1
TRINITY_DN64408_c0_g1_i1	17.4 kDa class III heat shock protein	20	10	57	38	41	42	43	313
TRINITY_DN64408_c0_g1_i2	17.4 kDa class III heat shock protein	2	6	11	2	4	6	6	72

TRINITY_DN64562_c0_g1_i3	wall-associated receptor kinase 3-like	8	4	17	15	0	4	13	1
TRINITY_DN64592_c0_g7_i3	probable LRR receptor-like serine/threonine-protein kinase	0	0	7	16	7	7	13	0
TRINITY_DN64609_c2_g12_i6	putative receptor-like protein kinase	25	27	37	0	56	26	22	33
TRINITY_DN64683_c0_g1_i1	LRR receptor-like serine threonine- kinase ERL1	1	12	10	7	3	10	26	2
TRINITY_DN64819_c1_g1_i2	late embryogenesis abundant protein	10	1	96	17	3	1	6	13
TRINITY_DN64848_c0_g1_i1	Peroxidase 12 precursor, putative	69	97	107	59	344	120	146	66
TRINITY_DN64848_c0_g2_i1	Peroxidase 12 precursor, putative	11	102	557	37	58	140	64	58
TRINITY_DN64882_c0_g7_i1	NBS-LRR disease resistance	4	0	0	0	0	25	0	0
TRINITY_DN64940_c0_g2_i2	methylthioribose kinase	27	9	5	7	10	3	4	4
TRINITY_DN65026_c0_g11_i1	LRR receptor-like serine threonine- kinase GSO1	13	127	0	0	25	0	0	0
TRINITY_DN65192_c1_g6_i1	NBS-containing resistance	119	73	0	0	0	0	0	0
TRINITY_DN65201_c1_g4_i1	uncharacterized serine-rich protein C215.13	37	27	49	22	36	18	16	55
TRINITY_DN65322_c0_g1_i1	probable LRR receptor-like serine threonine- kinase	88	0	37	7	0	0	0	12
TRINITY_DN65330_c2_g1_i4	SNF1-related protein kinase regulatory subunit beta-1-like	4	5	4	4	5	4	7	9
TRINITY_DN65498_c0_g2_i1	Putative tify domain/CCT motif transcription factor	145	303	231	187	281	345	357	147
TRINITY_DN65711_c0_g2_i1	CBL-interacting serine/threonine-protein kinase 12-like	7	4	3	2	2	1	1	5
TRINITY_DN65925_c0_g1_i1	predicted protein	7	5	4	3	7	2	1	2
TRINITY_DN65963_c0_g1_i1	serine threonine- kinase UCNL	2	7	10	15	6	10	22	2
TRINITY_DN65974_c0_g1_i3	RR4 - Corn type-A response regulator	25	61	59	56	54	11	44	29
TRINITY_DN66018_c0_g1_i2	aleurain-like	39	10	16	1722	26	5224	8	11500
TRINITY_DN66141_c0_g2_i2	60S ribosomal protein L31	96	247	105	121	134	175	228	120
TRINITY_DN66195_c0_g2_i1	Ascorbate peroxidase	16	11	13	16	16	21	10	26
TRINITY_DN66195_c0_g2_i2	Ascorbate peroxidase	77	36	0	0	14	26	16	31
TRINITY_DN66234_c0_g3_i1	cc-nbs-lrr resistance protein	4	4	2	21	19	0	0	0
TRINITY_DN66268_c0_g1_i1	putative wall-associated receptor kinase-like 16	8	1	0	4	2	0	1	4
TRINITY_DN66429_c0_g2_i1	methylthioribose kinase	10	38	16	4	7	0	0	5
TRINITY_DN66510_c0_g1_i1	LRR receptor-like serine/threonine-protein kinase GSO1	3	27	15	32	30	60	14	19
TRINITY_DN66518_c0_g6_i2	late embryogenesis abundant Lea5-like	0	0	0	0	0	46	24	0
TRINITY_DN66525_c0_g3_i1	kDa class I heat shock -like	14	29	81	18	41	49	41	438
TRINITY_DN66545_c0_g1_i1	transcription factor MYB44-like	0	4	1	1	1	6	24	0
TRINITY_DN66676_c0_g1_i2	peroxidase 1-like	8	78	50	76	23	44	176	16
TRINITY_DN66686_c0_g14_i1	putative rust resistance kinase Lr10	2	8	10	2	16	8	2	3
TRINITY_DN66697_c2_g3_i1	probable NADH dehydrogenase [ubiquinone]	46	68	46	34	44	72	43	33

TRINITY_DN66697_c2_g3_i3	probable NADH dehydrogenase [ubiquinone]	17	0	31	35	14	0	15	27
TRINITY_DN66721_c1_g1_i1	Uridylate kinase	67	103	47	58	86	62	108	41
TRINITY_DN66874_c3_g2_i2	peptide methionine sulfoxide reductase B5-like	43	30	33	40	41	68	30	88
TRINITY_DN66903_c0_g1_i2	nuclear transcription factor Y subunit B-1-like	6	35	13	21	8	10	11	3
TRINITY_DN66933_c0_g2_i2	U-box domain-containing protein 33-like	7	3	0	3	1	1	0	3
TRINITY_DN66933_c0_g2_i3	U-box domain-containing protein 33-like	8	4	6	5	1	2	3	3
TRINITY_DN66933_c0_g2_i4	U-box domain-containing 33-like isoform X1	0	8	8	0	9	12	6	9
TRINITY_DN66933_c0_g2_i5	U-box domain-containing 33-like	8	0	3	0	0	0	3	0
TRINITY_DN67087_c0_g1_i3	photosynthetic NDH subunit of subcomplex B chloroplastic	4	6	8	13	11	19	2	17
TRINITY_DN67087_c0_g1_i7	predicted protein	144	76	97	115	114	104	38	100
TRINITY_DN67088_c0_g7_i2	kDa class V heat shock	3	5	3	3	1	30	45	81
TRINITY_DN67102_c0_g1_i2	putative peroxidase 49 precursor	14	59	27	11	22	36	50	25
TRINITY_DN67203_c1_g1_i1	adenylate kinase B-like	13	2	9	6	9	19	6	8
TRINITY_DN67312_c0_g1_i1	peroxidase 43	8	28	36	43	4	26	99	8
TRINITY_DN67434_c0_g1_i2	peroxidase 72	0	7	3	7	2	7	16	1
TRINITY_DN67434_c0_g1_i3	peroxidase 72-like	1	0	2	0	0	5	0	0
TRINITY_DN67438_c0_g1_i1	probable leucine-rich repeat receptor-like protein kinase	4	23	24	41	40	48	25	29
TRINITY_DN67458_c4_g2_i1	RR4 - Corn type-A response regulator	3	13	4	13	11	3	7	6
TRINITY_DN67463_c3_g5_i7	ethylene-responsive transcription factor RAP2-12-like	3	0	0	0	1	1	1	49
TRINITY_DN67584_c0_g1_i1	zinc finger protein ZAT10-like	61	45	202	57	52	110	90	50
TRINITY_DN67698_c0_g1_i2	phosphopantetheine adenylyltransferase	5	5	2	1	6	6	4	3
TRINITY_DN67748_c0_g4_i1	peroxidase 4	8	38	44	20	10	25	121	1
TRINITY_DN67766_c1_g1_i3	transcription factor bHLH128-like	28	20	25	25	23	7	7	11
TRINITY_DN67799_c0_g1_i1	heat shock 70 kDa protein, mitochondrial-like	38	56	41	18	33	77	74	138
TRINITY_DN67803_c0_g1_i1	OAY73438.1Wall-associated receptor kinase 2	68	0	35	56	35	303	64	0
TRINITY_DN67906_c0_g1_i1	cationic peroxidase 1-like	14	28	22	19	20	17	12	9
TRINITY_DN67968_c0_g14_i1	probable LRR receptor-like serine/threonine-protein kinase	65	8	0	0	7	0	0	12
TRINITY_DN67968_c0_g19_i1	predicted protein	104	80	61	175	115	10	7	61
TRINITY_DN67984_c1_g2_i1	calcium-dependent protein kinase CPK1 adapter protein 2-like	3	6	5	4	6	6	7	5
TRINITY_DN68010_c0_g1_i2	predicted protein	145	214	68	22	8	205	3	112
TRINITY_DN68055_c0_g1_i1	Peroxidase 16	0	21	5	8	0	11	16	0
TRINITY_DN68055_c0_g2_i1	Peroxidase 16	0	14	4	4	2	5	15	1
TRINITY_DN68131_c2_g1_i1	transcription factor bHLH35	2	1	4	0	3	8	19	6

TRINITY_DN68238_c0_g1_i1	glycine-rich RNA binding protein	110	256	197	174	208	138	280	123
TRINITY_DN68238_c0_g3_i2	glycine-rich RNA binding protein	520	506	412	1384	355	832	762	566
TRINITY_DN68246_c0_g1_i1	serine threonine- kinase CCR3	3	2	19	6	5	12	9	7
TRINITY_DN68273_c0_g1_i4	MAP Kinase	63	49	71	38	65	47	39	47
TRINITY_DN68273_c0_g2_i1	MAP kinase 5	21	19	19	22	25	8	17	42
TRINITY_DN68273_c0_g3_i1	MAP kinase 5	7	3	0	22	0	12	2	2
TRINITY_DN68273_c0_g4_i3	MAP Kinase	28	25	36	29	27	27	23	21
TRINITY_DN68273_c0_g8_i1	MAP kinase 5	29	21	33	9	20	24	16	7
TRINITY_DN68275_c0_g1_i2	predicted protein	2	4	3	8	0	23	1	2
TRINITY_DN68368_c2_g14_i1	LRR receptor-like serine threonine- kinase GSO1	68	6	0	19	0	9	0	0
TRINITY_DN68439_c1_g3_i1	probable calcium-binding protein CML13-like	84	65	86	72	91	68	84	68
TRINITY_DN68563_c2_g1_i2	kDa heat shock -like	3	7	4	41	12	44	8	682
TRINITY_DN68666_c0_g2_i1	probable LRR receptor-like serine threonine- kinase	0	0	0	0	256	78	26	0
TRINITY_DN68737_c0_g3_i2	p21-activated protein kinase-interacting protein 1-like	5	5	6	6	4	10	9	11
TRINITY_DN68800_c1_g7_i1	MDIS1-interacting receptor like kinase 2	226	208	113	143	257	127	80	338
TRINITY_DN68814_c0_g5_i1	peroxidase P7-like	10	62	48	73	17	65	209	18
TRINITY_DN68894_c0_g1_i1	predicted protein	4	5	3	3	3	3	3	4
TRINITY_DN68894_c0_g1_i2	glyoxylate/succinic semialdehyde reductase 1	86	85	102	64	99	58	56	63
TRINITY_DN68894_c0_g1_i3	predicted protein	21	8	6	4	19	13	1	8
TRINITY_DN69044_c0_g3_i1	catalase	241	1175	1116	246	1106	1844	1746	4364
TRINITY_DN69045_c2_g10_i1	retrotransposon unclassified	0	0	0	359	0	2238	24	29
TRINITY_DN69045_c2_g2_i2	retrotransposon unclassified	0	0	0	0	0	24	0	39
TRINITY_DN69047_c0_g1_i1	predicted protein	105	128	155	123	132	134	163	89
TRINITY_DN69101_c0_g10_i2	mevalonate kinase isoform X1	4	4	1	6	1	8	4	3
TRINITY_DN69101_c0_g3_i2	Mevalonate kinase	4	4	5	5	3	11	6	5
TRINITY_DN69113_c0_g4_i4	16.0 kDa heat shock protein, peroxisomal	5	6	8	11	11	25	8	81
TRINITY_DN69129_c0_g1_i1	DNAJ heat shock N-terminal domain-containing protein	76	35	34	29	39	149	67	128
TRINITY_DN69274_c0_g1_i1	predicted protein	18	19	17	19	25	21	17	22
TRINITY_DN69296_c0_g3_i5	predicted protein	28	16	23	5	14	27	14	12
TRINITY_DN69303_c1_g2_i1	phytosulfokine receptor 1-like	22	0	265	0	0	0	80	97
TRINITY_DN69303_c1_g7_i1	phytosulfokine receptor 1-like	17	257	125	309	97	104	121	160
TRINITY_DN69316_c1_g1_i2	heat shock protein 90	1	28	16	26	19	41	24	417
TRINITY_DN69332_c0_g3_i4	Serine/arginine-rich splicing factor RS2Z33	127	175	132	117	170	43	85	48

TRINITY_DN69369_c0_g1_i1	probable LRR receptor-like serine threonine- kinase IRK	0	13	5	7	1	7	19	0
TRINITY_DN69382_c0_g5_i1	heat shock protein 70	50	164	93	51	7	208	246	493
TRINITY_DN69450_c1_g1_i1	probable amino-acid acetyltransferase chloroplastic	1	0	1	3	0	9	6	5
TRINITY_DN69457_c0_g1_i1	methylthioribose kinase-like	15	23	2	10	1	0	1	0
TRINITY_DN69476_c3_g5_i3	GTP pyrophosphokinase-like	9	4	5	3	8	3	1	6
TRINITY_DN69478_c0_g1_i1	peroxidase 52-like	9	31	58	27	5	18	59	2
TRINITY_DN69478_c0_g2_i1	putative bacterial-induced peroxidase precursor	7	8	6	4	1	6	18	1
TRINITY_DN69486_c0_g1_i1	Protein IRX15-LIKE	20	39	31	42	17	31	44	26
TRINITY_DN69637_c1_g1_i2	DAG protein, chloroplastic-like	8	16	20	26	16	68	17	22
TRINITY_DN69681_c1_g3_i1	ethylene receptor	19	10	23	17	11	21	27	20
TRINITY_DN69691_c0_g1_i1	peroxidase 19-like	0	6	7	11	3	9	32	1
TRINITY_DN69783_c1_g4_i1	predicted protein	43	0	23	4	14	40	4	49
TRINITY_DN69806_c2_g1_i13	diacylglycerol kinase 5-like	8	2	6	0	1	0	8	4
TRINITY_DN69821_c1_g15_i1	peroxidase 73-like	6	7	13	8	1	1	4	8
TRINITY_DN69821_c1_g15_i3	peroxidase 51-like	4	4	0	6	5	2	6	0
TRINITY_DN69855_c1_g1_i1	Pto kinase interactor	10	14	0	31	14	9	4	0
TRINITY_DN69855_c1_g9_i1	Pto kinase interactor	9	6	16	21	4	10	7	2
TRINITY_DN69886_c2_g1_i1	probable fructose-bisphosphate aldolase 3, chloroplastic-like	84	236	159	164	149	241	326	128
TRINITY_DN69886_c2_g1_i2	predicted protein	10	9	17	14	10	13	55	16
TRINITY_DN69896_c0_g2_i1	ICE-like protease p20 domain containing protein	8	6	9	8	7	6	7	7
TRINITY_DN69924_c0_g1_i1	GTP pyrophosphokinase	9	7	5	5	4	3	5	8
TRINITY_DN70138_c1_g1_i4	cysteine inhibitor 1	47	29	51	28	48	62	36	58
TRINITY_DN70254_c2_g20_i1	NBS-LRR resistance	0	0	0	0	0	80	0	0
TRINITY_DN70308_c5_g5_i1	predicted protein	60	87	20	41	58	77	99	74
TRINITY_DN70352_c0_g1_i1	Serine/threonine-protein kinase BLUS1	13	6	9	4	14	6	2	5
TRINITY_DN70390_c3_g3_i1	probable adenylate kinase 1, chloroplastic isoform X1	17	12	6	19	16	10	7	6
TRINITY_DN70418_c0_g2_i2	BAF09208.1 Os02g0592500	1	0	5	1	1	6	2	3
TRINITY_DN70424_c0_g3_i1	SKP1-like protein 1B	125	191	140	201	170	162	219	156
TRINITY_DN70433_c1_g1_i1	3-beta hydroxysteroid dehydrogenase/isomerase family protein	56	29	33	27	37	45	29	59
TRINITY_DN70452_c1_g2_i3	Peroxidase 52 precursor, putative	5	7	3	3	11	1	1	8
TRINITY_DN70469_c0_g1_i3	CAH65763.1 H0215A08.5	16	11	13	14	35	5	3	8
TRINITY_DN70500_c0_g1_i1	Peroxidase 1	18	55	57	39	30	24	29	12
TRINITY_DN70500_c0_g4_i1	Peroxidase 1	17	131	88	64	45	33	59	26

TRINITY_DN70576_c0_g1_i2	aarF domain-containing protein kinase, chloroplastic-like	3	1	1	1	0	0	0	1
TRINITY_DN70618_c0_g4_i1	predicted protein	86	99	116	72	29	471	109	132
TRINITY_DN70669_c1_g2_i1	predicted protein	23	20	13	17	26	28	13	20
TRINITY_DN70696_c1_g15_i1	predicted protein	6	5	0	0	1	3	2	10
TRINITY_DN70734_c1_g3_i1	predicted protein	11	1	7	5	2	2	4	24
TRINITY_DN70734_c1_g3_i2	predicted protein	39	5	2	7	5	7	2	25
TRINITY_DN70789_c0_g2_i5	heat shock 83	13	27	16	32	35	42	30	862
TRINITY_DN70819_c1_g2_i2	glutathione peroxidase 4	12	11	20	13	19	27	23	32
TRINITY_DN70928_c0_g12_i1	probable receptor-like protein kinase	0	0	0	7	5	17	0	0
TRINITY_DN70930_c2_g5_i1	Serine threonine- kinase HT1	4	21	0	42	0	0	0	0
TRINITY_DN71076_c0_g2_i6	predicted protein	0	69	0	19	44	5	21	61
TRINITY_DN71109_c2_g1_i11	Aspartokinase 1, chloroplastic	3	0	1	7	6	2	3	2
TRINITY_DN71109_c2_g1_i6	aspartokinase 2, chloroplastic-like	6	4	4	2	3	2	3	3
TRINITY_DN71180_c0_g11_i1	probable LRR receptor-like serine/threonine-protein kinase	357	119	43	223	294	100	78	163
TRINITY_DN71299_c1_g1_i2	uncharacterized bolA-like protein C8C9.11-like	231	264	229	182	254	243	201	218
TRINITY_DN71336_c0_g1_i1	serine/threonine-protein kinase OXI1-like	17	20	20	16	17	12	16	7
TRINITY_DN71337_c0_g1_i1	kDa class II heat shock -like	2	8	3	10	7	20	4	519
TRINITY_DN71459_c0_g5_i1	retrotransposon unclassified	3	2	6	10	6	4	6	185
TRINITY_DN71761_c0_g2_i2	peroxidase 16-like	15	21	32	50	37	17	59	16
TRINITY_DN71768_c0_g2_i2	putative uridine kinase C227.14-like	5	6	6	0	6	5	1	3
TRINITY_DN71768_c0_g2_i3	putative uridine kinase C227.14-like	2	9	5	9	7	8	11	14
TRINITY_DN71768_c0_g3_i1	putative uridine kinase C227.14-like	3	12	9	34	8	7	8	21
TRINITY_DN71768_c0_g4_i2	putative uridine kinase C227.14-like	2	4	1	0	0	5	1	0
TRINITY_DN71768_c0_g4_i5	putative uridine kinase C227.14	9	12	2	11	11	9	4	10
TRINITY_DN71792_c2_g6_i1	predicted protein	33	0	11	18	84	59	59	47
TRINITY_DN71792_c2_g7_i9	predicted protein	7	1	7	2	4	19	3	5
TRINITY_DN71917_c0_g2_i1	SNF1-related protein kinase regulatory subunit beta-3-like	17	13	14	12	15	14	15	10
TRINITY_DN72104_c1_g1_i6	Receptor-like kinase LIP2	0	3	0	0	4	3	5	2
TRINITY_DN72137_c0_g2_i2	TOM1 2	46	26	7	0	23	77	0	32
TRINITY_DN72163_c1_g3_i4	predicted protein	2	6	2	6	2	5	2	3
TRINITY_DN72327_c0_g2_i1	inactive leucine-rich repeat receptor-like serine threonine-kinase	1	4	2	1	1	5	6	0
TRINITY_DN72377_c0_g2_i1	zinc finger protein	261	193	3	308	3	2	43	118
TRINITY_DN72444_c2_g1_i2	somatic embryogenesis receptor kinase 1-like	14	18	14	12	8	5	12	5

TRINITY_DN72444_c2_g2_i6	brassinosteroid insensitive 1-associated receptor kinase 1 precursor	11	19	13	14	8	4	21	3
TRINITY_DN72460_c0_g1_i3	Receptor protein kinase-like protein	8	0	4	0	0	0	0	42
TRINITY_DN72566_c0_g18_i1	wall-associated receptor kinase 2-like	131	0	0	0	0	0	0	0
TRINITY_DN72566_c0_g1_i4	Wall-associated receptor kinase 3	51	3	29	47	0	20	13	3
TRINITY_DN72566_c0_g22_i1	putative wall-associated receptor kinase-like 16	28	7	17	14	10	50	15	4
TRINITY_DN72566_c0_g24_i3	putative wall-associated receptor kinase-like 16	27	10	10	15	19	14	11	10
TRINITY_DN72566_c0_g9_i1	Wall-associated receptor kinase 3	10	5	10	16	16	26	15	10
TRINITY_DN72595_c0_g1_i1	thymidine kinase-like	24	6	10	10	9	3	2	10
TRINITY_DN72595_c0_g6_i1	predicted protein	4	4	1	1	1	8	2	2
TRINITY_DN72595_c0_g6_i2	predicted protein	4	3	4	4	9	6	6	11
TRINITY_DN72595_c0_g7_i2	thymidine kinase-like	14	12	5	13	12	11	11	12
TRINITY_DN72604_c2_g2_i2	predicted protein	229	266	238	208	195	215	161	285
TRINITY_DN72604_c2_g5_i1	brassinosteroid insensitive 1-associated receptor kinase 1 precursor	55	16	19	54	34	22	0	29
TRINITY_DN72604_c2_g5_i3	brassinosteroid insensitive 1-associated receptor kinase 1 precursor	115	95	97	84	102	78	87	90
TRINITY_DN72607_c0_g14_i1	ATP DNA binding	7	18	11	28	18	16	7	29
TRINITY_DN72614_c0_g1_i1	Heat stress transcription factor A-3	12	2	5	6	7	15	4	90
TRINITY_DN72684_c1_g1_i2	peroxidase 3	14	56	41	22	10	46	108	10
TRINITY_DN72686_c1_g1_i3	probable adenylate kinase 1, chloroplastic-like	8	29	10	20	10	21	30	11
TRINITY_DN72705_c0_g1_i4	N utilization substance protein B homolog	28	13	19	19	15	83	27	49
TRINITY_DN72721_c0_g2_i5	CBL-interacting serine threonine- kinase 3 isoform X1	16	8	8	7	8	14	7	7
TRINITY_DN72738_c0_g1_i3	serine/threonine-protein kinase EDR1-like	5	6	4	3	0	1	3	3
TRINITY_DN72828_c0_g1_i1	hydroxyethylthiazole kinase-like	2	0	5	9	7	5	3	14
TRINITY_DN72828_c0_g7_i2	hydroxyethylthiazole kinase-like	6	0	3	1	1	2	0	0
TRINITY_DN72849_c0_g1_i1	leucine-rich repeat extensin-like protein 6-like	4	12	11	10	3	12	10	4
TRINITY_DN72875_c1_g5_i4	small heat shock protein	3	6	38	10	11	24	1	636
TRINITY_DN72875_c1_g5_i9	heat shock HSP26	133	294	506	405	302	420	408	8601
TRINITY_DN72877_c2_g6_i1	Heat shock cognate 70 kDa protein 1	196	131	108	78	129	266	591	165
TRINITY_DN72888_c0_g1_i1	peroxidase 4-like	0	2	2	1	4	18	10	2
TRINITY_DN72908_c0_g7_i1	heme oxygenase chloroplastic-like	117	0	175	41	127	226	89	0
TRINITY_DN72931_c1_g3_i2	2-cys peroxiredoxin-like protein	234	175	182	179	251	215	114	253
TRINITY_DN73024_c2_g1_i1	Tripeptidyl-peptidase 2	4	4	4	4	6	3	6	5

TRINITY_DN73066_c1_g3_i2	probable choline kinase 2	4	4	2	5	3	1	3	2
TRINITY_DN73066_c1_g3_i8	probable choline kinase 2	20	24	22	18	22	17	31	9
TRINITY_DN73083_c1_g3_i1	LRR receptor-like serine threonine- kinase GSO1	4	7	7	8	3	5	14	2
TRINITY_DN73139_c0_g2_i3	probable serine threonine- kinase	3	4	0	2	2	4	1	3
TRINITY_DN73141_c0_g2_i1	ATP binding	39	48	35	39	39	77	62	83
TRINITY_DN73262_c4_g1_i2	rop guanine nucleotide exchange factor 3	5	2	0	4	3	2	3	1
TRINITY_DN73343_c0_g2_i7	cyclin-T1-5-like isoform X1	4	2	4	4	3	3	5	4
TRINITY_DN73412_c0_g1_i3	rust resistance kinase Lr10-like	3	2	5	5	0	0	3	2
TRINITY_DN73463_c1_g1_i1	kDa class I heat shock -like	35	31	98	471	154	437	102	6069
TRINITY_DN73478_c1_g2_i2	uridylate kinase	25	11	16	22	15	34	17	34
TRINITY_DN73503_c3_g2_i5	protein phosphatase 1 regulatory subunit 7	7	7	7	9	10	9	9	7
TRINITY_DN73503_c3_g2_i8	protein phosphatase 1 regulatory subunit 7	3	4	4	6	4	3	6	6
TRINITY_DN73559_c3_g1_i1	dephospho-CoA kinase	6	8	1	2	5	15	6	8
TRINITY_DN73559_c3_g1_i2	dephospho-CoA kinase domain-containing protein-like	53	33	25	54	32	47	27	36
TRINITY_DN73559_c3_g1_i6	dephospho-CoA kinase	5	3	3	1	2	1	0	1
TRINITY_DN73561_c1_g1_i2	Ascorbate peroxidase	10	17	2	8	5	9	7	2
TRINITY_DN73582_c0_g1_i1	phosphatidylinositol 4-kinase gamma 5-like	3	5	7	9	5	6	15	11
TRINITY_DN73670_c0_g5_i1	probable ornithine aminotransferase-like	1	2	5	1	2	25	4	2
TRINITY_DN73705_c2_g1_i1	probable LRR receptor-like serine threonine- kinase	9	19	11	15	9	14	38	6
TRINITY_DN73722_c1_g4_i1	kinase associated protein phosphatase	21	29	17	33	26	21	19	8
TRINITY_DN73788_c5_g19_i1	Disease resistance protein (CC-NBS-LRR)	63	0	0	0	0	48	41	6
TRINITY_DN73826_c1_g1_i1	putative UPF0481 protein	7	1	4	3	2	5	6	3
TRINITY_DN73840_c0_g1_i1	zinc finger constans-like 2-like	41	55	58	60	39	17	12	9
TRINITY_DN73953_c2_g3_i2	WD repeat domain phosphoinositide-interacting protein 3-like	9	7	11	13	6	10	8	10
TRINITY_DN73979_c0_g1_i1	peroxidase A2-like	0	3	0	0	1	13	1	0
TRINITY_DN73984_c1_g4_i1	predicted protein	23	3	17	0	27	15	0	9
TRINITY_DN73984_c1_g7_i1	predicted protein	37	18	36	19	16	10	6	10
TRINITY_DN74141_c1_g1_i5	serine threonine- kinase STY8	8	6	3	6	5	4	4	5
TRINITY_DN74141_c1_g2_i1	serine/threonine-protein kinase STY46-like isoform X4	19	18	9	16	13	17	10	17
TRINITY_DN74216_c0_g5_i1	Wall-associated receptor kinase 3	62	33	15	24	30	23	14	18
TRINITY_DN74258_c0_g10_i1	NADH dehydrogenase [ubiquinone] flavo mitochondrial	0	39	23	0	23	36	11	113
TRINITY_DN74258_c0_g3_i1	NADH dehydrogenase [ubiquinone] flavoprotein 2, mitochondrial-like	52	59	212	127	236	228	241	290

TRINITY_DN74258_c0_g6_i2	predicted protein	13	10	9	0	21	20	32	0
TRINITY_DN74258_c0_g8_i2	predicted protein	34	58	19	27	30	53	87	30
TRINITY_DN74296_c0_g1_i2	casein kinase 1 HD16	4	0	1	1	0	0	0	0
TRINITY_DN74305_c0_g3_i3	putative serine/threonine kinase protein	8	9	8	12	3	6	12	4
TRINITY_DN74356_c0_g1_i1	probable adenylate kinase 7, mitochondrial	9	0	9	0	13	34	12	5
TRINITY_DN74356_c0_g3_i3	predicted protein	3	0	0	8	8	8	2	13
TRINITY_DN74357_c2_g3_i3	transcription initiation factor IIE subunit beta	12	22	22	90	33	32	27	1189
TRINITY_DN74366_c1_g4_i3	predicted protein	1	2	5	1	0	3	2	3
TRINITY_DN74394_c2_g1_i1	probable LRR receptor-like serine threonine- kinase	0	4	0	14	33	5	7	10
TRINITY_DN74394_c2_g5_i1	probable LRR receptor-like serine/threonine-protein kinase	11	12	49	5	5	68	10	46
TRINITY_DN74394_c2_g7_i1	probable LRR receptor-like serine threonine- kinase	0	0	0	0	11	7	0	8
TRINITY_DN74422_c0_g5_i1	dehydration responsive element binding protein 2 isoform c	92	42	99	76	60	112	78	363
TRINITY_DN74424_c2_g6_i5	Ribose-phosphate pyrophosphokinase 4	0	5	5	8	5	14	8	24
TRINITY_DN74432_c1_g1_i2	probable LRR receptor-like serine/threonine-protein kinase	35	0	7	0	14	0	0	8
TRINITY_DN74511_c0_g1_i5	heat shock factor HSF30-like	4	2	8	7	9	14	17	32
TRINITY_DN74700_c0_g4_i1	putative OsD305	4	9	4	40	8	27	8	5
TRINITY_DN74727_c1_g12_i1	peroxiredoxin- mitochondrial	0	71	214	188	63	200	233	93
TRINITY_DN74727_c1_g9_i1	peroxiredoxin- mitochondrial	0	36	18	7	0	29	11	3
TRINITY_DN74807_c1_g1_i4	probable choline kinase 2	25	18	22	27	18	40	18	43
TRINITY_DN74807_c1_g5_i2	probable choline kinase 2	7	6	5	10	2	8	14	16
TRINITY_DN74840_c2_g7_i5	probable NADH kinase	4	4	2	2	3	4	2	2
TRINITY_DN74848_c0_g3_i1	peroxidase P7-like	0	0	4	3	0	9	7	1
TRINITY_DN74889_c1_g2_i1	probable phospholipid hydroperoxide glutathione peroxidase	19	10	11	11	12	11	10	5
TRINITY_DN74892_c2_g1_i1	glutathione peroxidase	4	4	1	4	6	5	4	5
TRINITY_DN74917_c0_g3_i5	probable leucine-rich repeat receptor-like protein kinase At5g49770-like	4	2	2	2	1	5	3	3
TRINITY_DN74960_c1_g2_i1	IST1 homolog	60	104	93	123	0	194	282	34
TRINITY_DN74979_c1_g1_i4	diphosphomevalonate decarboxylase MVD2	2	10	4	8	4	7	14	4
TRINITY_DN74981_c4_g1_i1	oxidoreductase/ transition metal ion binding protein	10	4	8	4	5	5	6	23
TRINITY_DN74981_c4_g1_i2	oxidoreductase/ transition metal ion binding protein	10	7	15	7	2	9	5	18
TRINITY_DN74992_c1_g1_i1	bifunctional aspartokinase homoserine dehydrogenase chloroplastic	3	1	1	2	1	2	1	5
TRINITY_DN75009_c2_g2_i1	leucine-rich repeat receptor kinase PXC1	0	7	3	3	1	5	9	0

TRINITY_DN75061_c0_g7_i9	zinc finger BED domain-containing protein RICESLEEPER 2-like	2	3	105	4	24	6	4	5
TRINITY_DN75186_c1_g1_i1	probable L-ascorbate peroxidase 6, chloroplastic isoform X1	16	24	31	17	23	38	15	53
TRINITY_DN75308_c3_g3_i1	cysteine proteinase inhibitor A	0	52	0	32	0	14	7	32
TRINITY_DN75308_c3_g3_i2	cysteine proteinase inhibitor A	184	42	76	95	214	97	73	124
TRINITY_DN75308_c3_g3_i3	cysteine proteinase inhibitor 12-like isoform X2	213	53	77	102	169	136	55	141
TRINITY_DN75308_c3_g3_i4	cysteine proteinase inhibitor A	22	30	8	27	1	18	4	24
TRINITY_DN75308_c3_g4_i1	cysteine ase inhibitor A	37	0	55	29	0	0	21	2
TRINITY_DN75320_c0_g2_i2	putative 3,4-dihydroxy-2-butanone kinase	35	11	16	9	13	2	9	16
TRINITY_DN75347_c1_g4_i3	peroxidase 42	0	2	1	19	0	7	2	0
TRINITY_DN75347_c1_g4_i4	peroxidase 42	0	2	1	0	2	6	12	2
TRINITY_DN75349_c0_g1_i1	uridine-cytidine kinase C-like	1	4	2	3	3	5	2	3
TRINITY_DN75349_c0_g1_i3	uridine-cytidine kinase C-like	23	32	44	21	17	40	45	49
TRINITY_DN75349_c0_g1_i6	uridine-cytidine kinase C-like	13	10	10	12	8	14	14	14
TRINITY_DN75349_c0_g2_i1	uridine-cytidine kinase C isoform X2	4	2	4	1	1	6	5	10
TRINITY_DN75384_c1_g2_i17	uridine-cytidine kinase C-like	11	6	8	14	18	9	10	8
TRINITY_DN75553_c1_g5_i10	ONM08640.1Dihydroxyacetone kinase	10	5	6	11	11	7	5	12
TRINITY_DN75586_c1_g3_i1	OAY69846.1Thymidylate kinase	1	6	3	2	0	4	6	2
TRINITY_DN75586_c1_g3_i2	OAY69846.1Thymidylate kinase	13	11	3	8	13	17	19	17
TRINITY_DN75706_c2_g2_i5	nsp-interacting kinase 3	22	8	3	3	8	4	8	3
TRINITY_DN75761_c1_g2_i2	Glucosidase 2 subunit beta	9	15	8	9	8	15	17	7
TRINITY_DN75792_c0_g1_i1	predicted protein	3	3	3	2	4	6	2	6
TRINITY_DN75792_c0_g5_i1	predicted protein	4	4	3	1	4	4	1	8
TRINITY_DN75796_c0_g3_i1	peroxidase 72-like	0	0	1	4	0	3	16	0
TRINITY_DN75832_c2_g2_i11	Uridine kinase chloroplastic	3	7	8	6	11	8	11	11
TRINITY_DN75832_c2_g2_i12	uridine kinase-like protein 1, chloroplastic	5	4	0	7	3	2	0	6
TRINITY_DN75905_c3_g1_i15	methylmalonate-semialdehyde dehydrogenase [acylating] mitochondrial	1	15	3	0	0	8	0	6
TRINITY_DN75905_c3_g1_i6	methylmalonate-semialdehyde dehydrogenase [acylating] mitochondrial	15	6	5	8	9	10	9	7
TRINITY_DN76000_c1_g2_i1	shikimate kinase	12	7	8	5	11	10	5	9
TRINITY_DN76000_c1_g2_i10	shikimate kinase	3	0	6	1	3	11	7	5
TRINITY_DN76006_c1_g1_i7	dual specificity protein phosphatase Diacylglycerol kinase, catalytic region	4	3	5	3	0	1	3	1

TRINITY_DN76006_c1_g1_i9	dual specificity protein phosphatase Diacylglycerol kinase, catalytic region	4	4	3	4	0	6	4	4
TRINITY_DN76006_c1_g3_i2	dual specificity protein phosphatase Diacylglycerol kinase, catalytic region	4	1	3	2	4	6	4	4
TRINITY_DN76052_c0_g3_i1	predicted protein	10	13	16	13	15	17	24	8
TRINITY_DN76115_c1_g2_i3	rop guanine nucleotide exchange factor 3	23	12	14	16	20	21	17	30
TRINITY_DN76120_c1_g2_i1	dehydration-responsive element-binding protein 1C-like	0	0	0	0	0	50	13	0
TRINITY_DN76129_c0_g1_i4	Serine threonine- kinase 11-interacting	0	8	5	0	1	4	0	0
TRINITY_DN76129_c0_g1_i7	Serine threonine- kinase 11-interacting	3	8	4	7	5	5	9	1
TRINITY_DN76136_c1_g11_i1	B-box zinc finger 22-like	16	9	12	6	13	47	39	76
TRINITY_DN76244_c1_g5_i6	ascorbate peroxidase	105	118	15	84	78	0	112	91
TRINITY_DN76338_c5_g12_i1	probable GTP diphosphokinase RSH3, chloroplastic	3	2	0	0	1	2	0	10
TRINITY_DN76415_c0_g1_i1	glyceraldehyde-3-phosphate dehydrogenase-like protein	8	7	2	18	9	12	6	16
TRINITY_DN76423_c1_g5_i13	predicted protein	4	4	18	1	1	1	2	0
TRINITY_DN76443_c0_g6_i3	two-component response regulator ARR9-like	69	84	101	119	68	27	14	34
TRINITY_DN76503_c0_g13_i1	leaf rust 10 disease-resistance locus receptor-like protein kinase-like	171	48	115	58	154	67	91	62
TRINITY_DN76507_c2_g14_i3	probable LRR receptor-like serine/threonine-protein kinase	25	11	44	37	0	0	13	57
TRINITY_DN76507_c2_g17_i1	probable LRR receptor-like serine/threonine-protein kinase	39	10	53	34	19	22	3	13
TRINITY_DN76507_c2_g7_i1	probable LRR receptor-like serine/threonine-protein kinase	26	50	83	37	25	55	12	41
TRINITY_DN76558_c1_g3_i6	peroxidase P7-like	11	28	39	23	20	26	18	20
TRINITY_DN76592_c0_g13_i11	predicted protein	14	8	10	2	0	1	1	1
TRINITY_DN76634_c0_g2_i2	Serine/threonine protein kinase	5	1	1	0	0	0	0	2
TRINITY_DN76640_c3_g2_i3	pantothenate kinase	3	18	0	0	5	0	7	5
TRINITY_DN76640_c3_g2_i9	pantothenate kinase 4	4	4	1	2	5	2	8	0
TRINITY_DN76680_c0_g3_i2	probable amino-acid acetyltransferase NAGS1, chloroplastic	10	10	13	10	15	21	14	28
TRINITY_DN76699_c0_g5_i4	MOB kinase activator-like 1A	10	10	7	6	7	6	5	0
TRINITY_DN76699_c0_g5_i6	MOB kinase activator-like 1-like	7	5	7	7	6	12	11	16
TRINITY_DN76709_c2_g3_i5	diacylglycerol kinase 7-like	2	7	7	13	10	6	11	6
TRINITY_DN76709_c2_g3_i6	diacylglycerol kinase 7-like	13	17	17	29	15	13	16	15
TRINITY_DN76791_c1_g1_i4	predicted protein	10	5	12	24	15	12	10	9
TRINITY_DN76805_c0_g1_i2	aspartate/glutamate/uridylate kinase family protein	6	4	3	4	1	3	1	4
TRINITY_DN76805_c0_g1_i3	Glutamate 5-kinase	6	3	3	4	12	2	5	5
TRINITY_DN76805_c0_g2_i10	aspartate/glutamate/uridylate kinase family protein	5	5	8	4	5	4	6	2

TRINITY_DN76837_c2_g1_i3	ribose-phosphate pyrophosphokinase 5, chloroplastic-like	20	2	4	7	14	7	4	5
TRINITY_DN76837_c2_g1_i7	ribose-phosphate pyrophosphokinase 1	20	10	11	11	16	7	5	11
TRINITY_DN76857_c2_g6_i2	predicted protein	39	5	22	0	9	5	10	7
TRINITY_DN76869_c0_g3_i1	probable LRR receptor-like serine/threonine-protein kinase	58	50	52	47	40	46	41	40
TRINITY_DN76976_c2_g1_i16	homoserine kinase	4	8	5	8	7	11	11	3
TRINITY_DN76976_c2_g1_i18	homoserine kinase	3	6	0	8	6	11	4	0
TRINITY_DN76976_c2_g1_i5	homoserine kinase	27	70	31	46	41	77	80	23
TRINITY_DN76976_c2_g1_i6	homoserine kinase	13	52	31	51	39	90	37	13
TRINITY_DN77082_c1_g1_i1	probable phytol kinase 2, chloroplastic-like	18	13	16	15	14	15	21	15
TRINITY_DN77131_c0_g8_i1	probable LRR receptor-like serine/threonine-protein kinase	0	0	23	0	5	4	0	2
TRINITY_DN77180_c1_g20_i1	predicted protein	49	3	35	33	37	46	32	12
TRINITY_DN77180_c1_g4_i1	predicted protein	5	100	30	17	5	28	18	48
TRINITY_DN77189_c0_g6_i1	thiamine pyrophosphokinase 1 isoform X1	118	132	68	138	284	80	93	74
TRINITY_DN77189_c0_g9_i3	Thiamin pyrophosphokinase	8	12	7	11	27	11	10	8
TRINITY_DN77247_c0_g6_i1	ATP sulfurylase chloroplastic	289	335	136	172	132	181	134	279
TRINITY_DN77310_c1_g7_i1	phospholipid hydroperoxide glutathione peroxidase	15	19	11	13	13	23	17	12
TRINITY_DN77485_c0_g2_i1	receptor kinase-like protein Xa21	37	58	86	31	35	28	5	64
TRINITY_DN77485_c0_g3_i1	putative receptor-like protein kinase	80	0	0	0	0	15	0	7
TRINITY_DN77485_c0_g4_i1	putative receptor-like protein kinase	45	9	71	13	27	9	0	25
TRINITY_DN77485_c1_g15_i1	probable LRR receptor-like serine threonine- kinase	0	0	0	0	0	258	0	0
TRINITY_DN77573_c4_g10_i2	predicted protein	19	19	14	19	29	36	16	26
TRINITY_DN77573_c4_g3_i1	protein PLASTID REDOX INSENSITIVE 2-like	337	465	295	295	219	289	157	377
TRINITY_DN77577_c0_g2_i6	phosphoribulokinase, chloroplastic	431	279	330	219	459	207	82	272
TRINITY_DN77577_c0_g3_i2	phosphoribulokinase, chloroplastic	219	217	200	258	164	153	62	84
TRINITY_DN77577_c0_g4_i1	phosphoribulokinase, chloroplastic-like	913	58	356	246	879	209	64	508
TRINITY_DN77609_c2_g1_i2	Protein ABC1, mitochondrial precursor, putative	99	89	95	174	59	69	129	79
TRINITY_DN77625_c1_g1_i1	predicted protein	15	5	7	13	7	15	22	21
TRINITY_DN77633_c0_g3_i1	protein binding protein, putative	3	14	5	6	10	28	24	7
TRINITY_DN77679_c3_g2_i2	2-Cys peroxiredoxin BAS1, chloroplastic	129	144	102	143	145	163	105	197
TRINITY_DN77679_c3_g2_i5	2-Cys peroxiredoxin BAS1, chloroplastic	35	38	38	30	59	30	25	24
TRINITY_DN77679_c3_g3_i1	2-cys peroxiredoxin BAS1	35	62	135	0	57	0	14	0
TRINITY_DN77679_c3_g3_i2	2-cys peroxiredoxin BAS1	261	197	167	294	315	286	147	326
TRINITY_DN77679_c3_g3_i3	2-cys peroxiredoxin-like protein	25	16	14	16	20	13	12	14

TRINITY_DN77705_c3_g3_i5	aconitate hydratase, cytoplasmic-like	20	29	17	16	1	5	20	3
TRINITY_DN77733_c1_g6_i1	OEL37515.1Peroxidase 9	5	8	9	16	4	17	8	3
TRINITY_DN77735_c0_g9_i4	calcium/calmodulin-dependent serine/threonine-protein kinase 1-like	2	2	3	1	1	7	5	0
TRINITY_DN77810_c1_g3_i5	terpene synthase 10-like	179	77	0	0	0	0	11	0
TRINITY_DN77826_c0_g1_i1	probable LRR receptor-like serine threonine- kinase	5	4	5	5	6	5	3	6
TRINITY_DN77829_c0_g3_i5	Receptor-like serine threonine- kinase SD1-8	7	6	8	7	5	5	4	7
TRINITY_DN77830_c1_g2_i2	USP family protein	74	197	131	130	153	222	228	102
TRINITY_DN77832_c1_g13_i7	wall-associated receptor kinase 5-like	12	7	6	4	10	0	2	3
TRINITY_DN77884_c1_g1_i1	probable receptor kinase At1g11050	21	20	18	19	21	17	22	24
TRINITY_DN77945_c0_g6_i1	probable LRR receptor-like serine/threonine-protein kinase	136	78	35	97	111	28	20	33
TRINITY_DN78091_c0_g7_i1	peptide methionine sulfoxide reductase-like	61	75	68	73	79	109	49	111
TRINITY_DN78091_c0_g7_i3	peptide methionine sulfoxide reductase-like	61	21	38	4	29	49	34	48
TRINITY_DN78130_c1_g1_i1	protein FAM206A-like	33	25	24	17	24	13	19	14
TRINITY_DN78134_c1_g1_i1	Peptide methionine sulfoxide reductase msrB	3	4	5	6	3	5	0	17
TRINITY_DN78242_c0_g3_i1	proline-rich receptor-like protein kinase PERK3	5	2	2	2	2	3	2	3
TRINITY_DN78271_c0_g1_i8	water chloroplastic	41	33	38	25	44	44	41	54
TRINITY_DN78273_c1_g3_i3	UDP rhamnose:anthocyanidin-3-glucoside rhamnosyltransferase	5	2	2	2	5	12	12	14
TRINITY_DN78338_c0_g11_i1	phosphoenolpyruvate carboxykinase [ATP]-like	22	21	27	14	57	45	14	5
TRINITY_DN78338_c0_g13_i1	phosphoenolpyruvate carboxykinase [ATP]-like	15	5	89	18	111	45	11	100
TRINITY_DN78338_c0_g3_i1	phosphoenolpyruvate carboxykinase 4	483	119	192	325	357	50	117	140
TRINITY_DN78338_c0_g5_i1	phosphoenolpyruvate carboxykinase	124	69	31	0	0	35	4	52
TRINITY_DN78338_c0_g6_i1	phosphoenolpyruvate carboxykinase	637	552	257	389	314	176	190	139
TRINITY_DN78338_c0_g7_i1	phosphoenolpyruvate carboxykinase	163	142	196	229	194	68	107	125
TRINITY_DN78339_c1_g5_i1	kDa class I heat shock -like	84	70	442	79	92	325	183	2684
TRINITY_DN78340_c3_g7_i1	GATA transcription factor 26-like	10	0	0	0	5	31	21	19
TRINITY_DN78437_c2_g11_i1	Wall-associated receptor kinase 3	9	33	43	11	13	47	12	32
TRINITY_DN78437_c2_g12_i6	Wall-associated receptor kinase 3	15	10	34	18	8	4	17	20
TRINITY_DN78437_c2_g13_i1	Wall-associated receptor kinase 3	69	0	10	4	45	12	33	22
TRINITY_DN78439_c0_g1_i1	peptide methionine sulfoxide reductase B5-like	110	98	82	77	101	208	150	163
TRINITY_DN78439_c0_g2_i1	peptide methionine sulfoxide reductase msrB	47	58	32	46	51	91	56	67
TRINITY_DN78439_c0_g4_i1	Peptide methionine sulfoxide reductase B5	24	0	33	18	0	0	0	0
TRINITY_DN78491_c1_g1_i1	uridylate kinase	39	11	7	17	13	11	24	15

TRINITY_DN78491_c1_g2_i3	Uridylate kinase	3	2	4	6	4	3	7	2
TRINITY_DN78491_c1_g2_i4	uridylate kinase	3	7	2	7	2	5	6	2
TRINITY_DN78491_c1_g2_i7	Uridylate kinase	21	27	12	29	15	21	21	10
TRINITY_DN78491_c1_g3_i1	Uridylate kinase	47	51	41	31	9	36	25	29
TRINITY_DN78491_c1_g4_i1	uridylate kinase	7	34	29	14	13	29	38	17
TRINITY_DN78491_c1_g5_i1	UMP-CMP kinase 4	19	27	18	24	12	20	33	11
TRINITY_DN78505_c0_g4_i1	peptide methionine sulfoxide reductase chloroplastic	19	0	35	57	23	0	10	6
TRINITY_DN78518_c2_g10_i1	methylthioribose kinase 1-like	10	50	114	8	47	53	59	31
TRINITY_DN78518_c2_g13_i1	methylthioribose kinase 1-like	81	74	70	53	62	63	77	86
TRINITY_DN78518_c2_g13_i2	Methylthioribose kinase	121	81	27	34	24	27	13	9
TRINITY_DN78518_c2_g13_i4	Methylthioribose kinase	159	68	49	50	12	48	45	13
TRINITY_DN78518_c2_g15_i1	methylthioribose kinase	74	56	52	52	43	91	68	104
TRINITY_DN78518_c2_g15_i2	methylthioribose kinase	52	41	10	0	33	24	15	18
TRINITY_DN78518_c2_g17_i1	methylthioribose kinase-like	24	4	14	23	33	11	12	21
TRINITY_DN78518_c2_g18_i1	methylthioribose kinase 1-like	7	7	3	0	10	0	18	32
TRINITY_DN78518_c2_g19_i2	methylthioribose kinase-like	146	88	0	0	50	29	0	5
TRINITY_DN78518_c2_g21_i1	5-methylthioribose kinase family protein	126	68	30	44	8	15	15	25
TRINITY_DN78518_c2_g2_i1	methylthioribose kinase	454	278	78	130	73	109	56	71
TRINITY_DN78518_c2_g4_i1	KZV45982.1methylthioribose kinase	103	89	27	11	20	5	12	14
TRINITY_DN78518_c2_g6_i1	methylthioribose kinase	77	48	0	10	9	48	1	0
TRINITY_DN78518_c2_g7_i1	methylthioribose kinase	17	51	3	17	0	0	0	3
TRINITY_DN78526_c1_g10_i1	dual specificity protein phosphatase 1B-like isoform X2	91	80	50	59	67	81	45	73
TRINITY_DN78527_c1_g1_i1	probable inactive receptor kinase	2	13	9	15	3	9	30	2
TRINITY_DN78544_c1_g9_i1	heat shock	19	27	43	1	37	42	5	628
TRINITY_DN78579_c0_g5_i1	putative proline-rich receptor-like protein kinase PERK11	12	45	29	40	17	16	11	3
TRINITY_DN78654_c0_g11_i1	probable LRR receptor-like serine/threonine-protein kinase	4	9	12	10	8	4	9	5
TRINITY_DN78683_c0_g4_i5	OAY75725.1U-box domain-containing protein 33	2	5	4	3	4	4	5	2
TRINITY_DN78737_c2_g3_i2	phosphoglycerate kinase	6	0	1	1	2	1	1	2
TRINITY_DN78740_c4_g2_i7	uridine kinase-like protein 3-like	5	10	8	9	6	8	9	5
TRINITY_DN78740_c4_g4_i1	putative uracil phosphoribosyltransferase	7	0	9	0	17	14	8	0
TRINITY_DN78740_c4_g5_i1	putative uracil phosphoribosyltransferase	42	48	36	48	21	53	49	32
TRINITY_DN78771_c1_g1_i16	protein-ribulosamine 3-kinase, chloroplastic	20	2	0	4	11	0	0	2
TRINITY_DN78787_c2_g7_i4	cysteine-rich receptor-like protein kinase 25-like	23	4	2	7	2	2	4	6

TRINITY_DN78800_c1_g4_i12	uncharacterized aarF domain-containing kinase 1	8	2	7	6	6	4	3	2
TRINITY_DN78823_c0_g3_i6	folic acid synthesis protein fol1-like	71	58	24	45	35	50	26	43
TRINITY_DN78886_c0_g2_i1	N-acetyl-D-glucosamine kinase	0	2	5	6	4	9	5	0
TRINITY_DN78886_c0_g2_i10	N-acetyl-D-glucosamine kinase	22	9	21	10	27	12	6	17
TRINITY_DN78898_c2_g1_i4	GAV92666.1zf-B_box domain-containing protein	19	13	16	11	21	4	4	1
TRINITY_DN78909_c1_g1_i6	putative disease resistance protein RGA3	3	15	1	1	9	5	21	7
TRINITY_DN78918_c0_g3_i8	adenosine kinase 2	183	390	237	192	186	418	536	276
TRINITY_DN78970_c1_g4_i1	cysteine-rich repeat secretory protein 38-like	5	5	1	3	1	0	18	3
TRINITY_DN79005_c1_g9_i3	uncharacterized aarF domain-containing protein kinase, chloroplastic-like	43	15	3	22	0	15	7	20
TRINITY_DN79005_c1_g9_i4	predicted protein	5	1	5	3	2	2	3	9
TRINITY_DN79005_c1_g9_i5	uncharacterized aarF domain-containing protein kinase, chloroplastic-like	8	14	27	14	26	22	9	16
TRINITY_DN79006_c2_g2_i1	NADH dehydrogenase [ubiquinone] iron-sulfur protein 1, mitochondrial-like	60	70	63	70	74	81	100	79
TRINITY_DN79009_c2_g9_i1	receptor-like serine threonine- kinase ALE2	0	6	12	12	12	12	11	18
TRINITY_DN79012_c1_g2_i1	L-type lectin-domain containing receptor kinase -like	1	5	3	2	0	4	10	1
TRINITY_DN79032_c1_g2_i2	predicted protein	64	37	25	19	60	22	3	15
TRINITY_DN79032_c1_g2_i9	adenylate kinase 5, chloroplastic-like isoform X2	19	6	0	3	8	1	1	1
TRINITY_DN79085_c2_g10_i1	aconitate hydratase, cytoplasmic-like	98	190	85	165	82	63	130	298
TRINITY_DN79085_c2_g9_i1	aconitate hydratase, cytoplasmic-like	55	67	82	101	73	39	61	30
TRINITY_DN79087_c2_g1_i7	phosphomevalonate kinase	3	9	7	5	2	4	3	7
TRINITY_DN79087_c2_g4_i2	phosphomevalonate kinase	8	2	0	0	0	0	3	5
TRINITY_DN79087_c2_g8_i1	phosphomevalonate kinase	23	28	27	36	39	39	33	26
TRINITY_DN79109_c0_g10_i1	Putative LRR receptor-like serine/threonine-protein kinase	26	19	25	22	5	10	5	21
TRINITY_DN79109_c0_g20_i1	predicted protein	60	70	5	44	15	58	77	67
TRINITY_DN79125_c3_g1_i1	probable GTP diphosphokinase RSH3, chloroplastic	5	1	1	1	1	0	1	0
TRINITY_DN79125_c3_g1_i15	GTP pyrophosphokinase	11	1	3	1	1	2	0	4
TRINITY_DN79163_c2_g1_i3	probable LRR receptor-like serine/threonine-protein kinase	3	39	11	12	4	7	33	1
TRINITY_DN79185_c0_g12_i1	phospholipid hydroperoxide glutathione peroxidase	295	558	446	368	379	382	486	285
TRINITY_DN79185_c0_g15_i2	phospholipid hydroperoxide glutathione peroxidase	762	572	427	602	613	319	273	281
TRINITY_DN79185_c0_g16_i1	phospholipid hydroperoxide glutathione peroxidase	246	446	351	298	317	357	319	243
TRINITY_DN79185_c0_g17_i2	probable phospholipid hydroperoxide glutathione peroxidase 6, mitochondrial-like	328	467	419	431	213	210	351	241

TRINITY_DN79185_c0_g1_i1	probable phospholipid hydroperoxide glutathione peroxidase	399	238	85	0	603	249	0	70
TRINITY_DN79185_c0_g4_i1	probable phospholipid hydroperoxide glutathione peroxidase 6, mitochondrial-like	199	542	339	375	341	473	529	254
TRINITY_DN79185_c0_g9_i1	glutathione peroxidase	78	33	125	47	505	69	96	105
TRINITY_DN79185_c0_g9_i2	glutathione peroxidase	306	201	150	220	104	73	78	63
TRINITY_DN79186_c0_g2_i1	receptor-like protein kinase HSL1	24	13	17	16	16	15	14	20
TRINITY_DN79214_c2_g1_i1	zinc-binding alcohol dehydrogenase domain-containing protein 2-like	110	130	190	134	123	205	107	116
TRINITY_DN79214_c2_g5_i13	zinc-binding alcohol dehydrogenase domain-containing protein 2-like	2	35	8	7	19	28	11	0
TRINITY_DN79214_c2_g5_i5	zinc-binding alcohol dehydrogenase domain-containing protein 2-like	12	4	20	7	18	17	13	5
TRINITY_DN79214_c2_g6_i2	ARP protein (REF)	33	55	40	74	45	49	25	58
TRINITY_DN79214_c2_g6_i3	15-hydroxyprostaglandin dehydrogenase	4	16	7	5	12	12	6	3
TRINITY_DN79214_c2_g6_i8	ARP protein (REF)	70	69	53	54	56	75	56	56
TRINITY_DN79243_c1_g1_i1	Pinus taeda anonymous locus CL314Contig1_03 genomic sequence	15	49	15	25	7	266	54	16
TRINITY_DN79243_c1_g2_i1	peroxidase 12-like	3	14	6	8	114	31	17	42
TRINITY_DN79243_c1_g5_i1	peroxidase 12-like	8	92	68	54	185	120	46	36
TRINITY_DN79243_c1_g6_i1	Pinus taeda anonymous locus CL314Contig1_03 genomic sequence	46	74	175	24	523	44	97	96
TRINITY_DN79243_c1_g6_i12	peroxidase 12	24	78	68	41	191	76	86	37
TRINITY_DN79243_c1_g6_i13	peroxidase	10	53	105	20	23	49	13	5
TRINITY_DN79243_c1_g6_i14	peroxidase 12	15	14	7	22	11	21	4	2
TRINITY_DN79243_c1_g6_i15	peroxidase 12-like	17	27	243	17	20	66	55	9
TRINITY_DN79243_c1_g6_i4	cationic peroxidase SPC4-like	26	43	23	30	122	65	31	62
TRINITY_DN79243_c1_g6_i5	peroxidase 12-like	6	16	8	8	28	18	6	15
TRINITY_DN79243_c1_g6_i8	peroxidase 12-like	16	24	82	38	19	36	10	5
TRINITY_DN79243_c1_g7_i1	peroxidase 12-like	0	0	11	0	95	76	20	82
TRINITY_DN79246_c1_g2_i1	wall-associated receptor kinase-like 16	4	3	2	3	3	3	2	5
TRINITY_DN79269_c2_g1_i9	transcription initiation factor TFIID subunit 7	5	0	0	0	2	2	10	30
TRINITY_DN79282_c1_g13_i1	receptor kinase HSL1	119	0	18	43	0	36	3	27
TRINITY_DN79282_c1_g16_i1	receptor kinase HSL1	77	0	6	56	4	11	3	6
TRINITY_DN79282_c1_g19_i1	receptor-like protein kinase HSL1	12	4	10	5	14	6	6	25

TRINITY_DN79282_c1_g2_i1	receptor-like protein kinase HSL1	262	134	123	186	92	77	42	53
TRINITY_DN79282_c1_g7_i1	receptor-like protein kinase HSL1	17	17	6	8	27	11	16	12
TRINITY_DN79300_c2_g5_i3	predicted protein	14	16	21	21	14	6	13	10
TRINITY_DN79300_c2_g5_i3	predicted protein	5	4	6	2	10	10	8	19
TRINITY_DN79300_c2_g5_i4	predicted protein	79	46	50	46	77	54	22	50
TRINITY_DN79367_c0_g5_i2	L-type lectin-domain containing receptor kinase S.4-like	5	3	3	1	2	9	8	2
TRINITY_DN79367_c0_g6_i1	L-type lectin-domain containing receptor kinase S.4-like	52	33	21	16	33	32	39	48
TRINITY_DN79393_c0_g3_i1	chaperone	92	129	493	199	176	390	425	2582
TRINITY_DN79399_c0_g1_i3	putative receptor protein kinase ZmPK1	35	39	211	28	103	28	32	19
TRINITY_DN79399_c0_g4_i1	receptor kinase 1	0	8	1	26	3	5	7	7
TRINITY_DN79399_c0_g9_i1	putative receptor protein kinase ZmPK1	34	31	14	8	43	24	25	27
TRINITY_DN79461_c6_g10_i1	MDIS1-interacting receptor like kinase 2-like	65	0	67	3	0	0	4	17
TRINITY_DN79461_c6_g10_i2	probable LRR receptor-like serine/threonine-protein kinase	21	22	20	47	34	34	45	80
TRINITY_DN79461_c6_g10_i3	MDIS1-interacting receptor like kinase 2-like	331	173	93	123	0	97	56	205
TRINITY_DN79461_c6_g10_i4	MDIS1-interacting receptor like kinase 2-like	33	0	0	0	46	14	45	0
TRINITY_DN79461_c6_g14_i2	probable leucine-rich repeat receptor-like protein kinase	9	0	0	0	0	0	0	47
TRINITY_DN79461_c6_g5_i1	MDIS1-interacting receptor like kinase 2-like	181	118	344	169	26	21	96	0
TRINITY_DN79467_c2_g1_i1	probable serine/threonine-protein kinase PBL1 isoform X2	5	10	2	0	2	21	0	2
TRINITY_DN79470_c1_g3_i2	serine/threonine-protein kinase AFC2 isoform X1	29	33	30	55	33	27	31	36
TRINITY_DN79470_c1_g8_i5	serine/threonine-protein kinase AFC2 isoform X1	97	65	74	64	79	43	78	46
TRINITY_DN79482_c1_g2_i1	proline responding1	17	12	32	23	0	50	29	46
TRINITY_DN79482_c1_g5_i1	Pyrroline-5-carboxylate synthetase isoform 3	7	8	7	15	3	7	5	9
TRINITY_DN79482_c1_g5_i2	Delta-1-pyrroline-5-carboxylate synthase	7	8	7	7	1	5	6	3
TRINITY_DN79482_c1_g5_i7	delta-1-pyrroline-5-carboxylate synthase-like isoform X3	21	34	26	42	25	30	40	17
TRINITY_DN79510_c2_g3_i3	putative serine/threonine-protein kinase isoform X1	8	10	3	7	9	5	9	7
TRINITY_DN79543_c0_g1_i11	Histidine kinase-like ATPase, C-terminal domain containing protein	3	7	5	4	0	4	6	5
TRINITY_DN79554_c0_g2_i1	GTP pyrophosphokinase	0	0	23	10	7	11	4	6
TRINITY_DN79554_c0_g2_i8	probable GTP diphosphokinase chloroplastic	15	11	13	13	12	10	10	14
TRINITY_DN79564_c0_g12_i1	class II small heat shock protein Le-HSP17.6	0	7	29	103	6	58	148	805
TRINITY_DN79564_c0_g1_i1	kDa class II heat shock -like	0	20	21	2	0	10	2	403
TRINITY_DN79565_c1_g2_i4	putative receptor-like protein kinase	5	2	0	3	7	3	1	2
TRINITY_DN79569_c2_g3_i2	protein MALE DISCOVERER 2	36	31	33	49	32	28	19	29
TRINITY_DN79569_c2_g3_i4	probable LRR receptor-like serine threonine- kinase MRH1	2	4	6	4	1	5	3	0

TRINITY_DN79573_c2_g1_i1	proline-rich receptor kinase PERK1	73	0	0	0	6	26	0	0
TRINITY_DN79583_c6_g7_i13	Wall-associated receptor kinase 5	33	38	18	20	22	25	37	60
TRINITY_DN79583_c6_g7_i17	Wall-associated receptor kinase 5	22	17	4	18	0	0	7	2
TRINITY_DN79599_c1_g11_i6	probable leucine-rich repeat receptor kinase	6	8	4	6	7	5	4	4
TRINITY_DN79599_c1_g1_i1	probable leucine-rich repeat receptor-like protein kinase	15	20	9	8	26	4	2	0
TRINITY_DN79620_c3_g3_i1	NAD kinase 1	380	269	409	425	360	209	225	518
TRINITY_DN79647_c1_g7_i1	Wall-associated receptor kinase 3	11	6	122	23	5	30	65	35
TRINITY_DN79647_c2_g2_i3	putative wall-associated receptor kinase-like 16	0	4	0	0	3	6	1	0
TRINITY_DN79725_c5_g8_i1	probable receptor-like protein kinase	54	23	64	18	21	50	29	34
TRINITY_DN79757_c0_g8_i2	adenylyl-sulfate kinase 3	8	8	8	6	6	9	9	6
TRINITY_DN79785_c0_g1_i1	retrotransposon unclassified	0	0	0	0	0	74	0	4
TRINITY_DN79785_c0_g2_i1	retrotransposon unclassified	4	0	4	46	0	216	0	4
TRINITY_DN79792_c8_g7_i1	putative leucine-rich repeat receptor-like protein kinase family protein	3	0	46	7	13	15	5	0
TRINITY_DN79794_c3_g3_i1	U-box domain-containing protein 33-like	18	18	10	22	23	28	21	26
TRINITY_DN79794_c3_g4_i1	U-box domain-containing protein 33-like	0	46	17	7	32	20	61	0
TRINITY_DN79794_c3_g5_i1	U-box domain-containing protein 33	37	58	63	53	25	77	38	17
TRINITY_DN79794_c3_g5_i2	receptor protein kinase, putative	9	102	19	30	9	29	50	30
TRINITY_DN79794_c4_g6_i1	U-box domain-containing protein 33-like	40	10	25	13	14	18	15	24
TRINITY_DN79804_c4_g5_i1	predicted protein	27	17	21	8	12	13	8	4
TRINITY_DN79854_c10_g5_i1	receptor 12	0	0	119	0	62	7	23	0
TRINITY_DN79857_c3_g3_i4	putative disease resistance protein Cf-2.1	15	6	3	2	0	6	2	1
TRINITY_DN79861_c1_g6_i1	predicted protein	14	5	8	10	14	3	2	3
TRINITY_DN79861_c1_g6_i4	OPAQUE10 isoform X1	6	7	8	8	5	1	0	5
TRINITY_DN79897_c1_g12_i2	putative leucine-rich repeat receptor-like protein kinase	28	74	31	72	22	56	8	66
TRINITY_DN79897_c1_g8_i1	putative leucine-rich repeat receptor-like protein kinase	21	22	0	47	3	8	3	39
TRINITY_DN79908_c4_g2_i1	probable LRR receptor-like serine/threonine-protein kinase	6	7	2	3	5	9	4	4
TRINITY_DN79939_c4_g29_i1	putative wall-associated kinase 4	81	40	10	23	40	22	7	30
TRINITY_DN79944_c6_g10_i1	retrotransposon unclassified	2	0	0	10	0	4	0	220
TRINITY_DN79944_c6_g6_i1	retrotransposon unclassified	0	0	0	0	0	21	0	368
TRINITY_DN79944_c6_g6_i2	retrotransposon unclassified	0	0	0	1	0	13	0	202
TRINITY_DN79944_c6_g6_i3	retrotransposon unclassified	5	0	0	207	2	641	2	1167
TRINITY_DN79944_c6_g9_i2	retrotransposon protein, putative, unclassified	3	0	0	1	0	10	0	243
TRINITY_DN79944_c6_g9_i6	retrotransposon protein, putative, unclassified	2	2	6	100	0	425	2	248

TRINITY_DN79950_c0_g1_i2	putative sugar kinase	10	17	14	11	10	16	18	19
TRINITY_DN79962_c3_g3_i16	heat stress transcription factor B-2a	1	1	10	5	2	3	3	91
TRINITY_DN79987_c1_g12_i1	probable leucine-rich repeat receptor kinase	14	0	18	38	11	10	6	18
TRINITY_DN79992_c10_g3_i4	serine-threonine protein kinase, plant-type, putative	0	0	0	1	0	13	0	0
TRINITY_DN79996_c5_g1_i1	ATPase subunit 1 (mitochondrion)	2683	4548	2512	2793	4447	3626	4706	3369
TRINITY_DN80011_c7_g2_i2	probable leucine-rich repeat receptor-like protein kinase	37	19	11	20	5	8	6	16
TRINITY_DN80014_c6_g3_i6	G-type lectin S-receptor-like serine threonine- kinase	4	3	1	0	3	1	0	0
TRINITY_DN80024_c7_g2_i1	probable LRR receptor-like serine threonine- kinase	638	166	160	95	123	201	126	149
TRINITY_DN80026_c0_g1_i1	L-type lectin-domain containing receptor kinase IV.1-like	54	21	4	3	49	21	27	28
TRINITY_DN80026_c1_g14_i3	lectin-like receptor kinase 7 isoform X1	56	21	2	11	53	41	41	22
TRINITY_DN80026_c1_g15_i1	L-type lectin-domain containing receptor kinase IV.1-like	36	0	4	4	21	7	4	60
TRINITY_DN80026_c1_g15_i2	L-type lectin-domain containing receptor kinase	0	37	22	36	11	26	40	119
TRINITY_DN80026_c1_g5_i1	L-type lectin-domain containing receptor kinase IV.1-like	11	11	24	18	30	16	6	22
TRINITY_DN80031_c13_g10_i1	Putative receptor-like protein kinase	3	6	10	5	5	30	15	1
TRINITY_DN80047_c6_g6_i2	probable receptor-like protein kinase	1	12	13	0	0	6	3	3
TRINITY_DN80049_c10_g4_i1	probable LRR receptor-like serine/threonine-protein kinase	8	5	0	3	6	8	1	5
TRINITY_DN80049_c10_g4_i4	probable LRR receptor-like serine threonine- kinase	5	6	4	3	6	11	4	4
TRINITY_DN80049_c10_g6_i1	probable LRR receptor-like serine/threonine-protein kinase	0	52	185	146	185	436	126	257
TRINITY_DN80049_c10_g7_i1	probable LRR receptor-like serine/threonine-protein kinase	11	40	11	19	53	60	0	27
TRINITY_DN80055_c1_g3_i1	predicted protein	65	7	19	16	0	10	12	14
TRINITY_DN80055_c1_g4_i9	Leucine-rich repeat receptor-like tyrosine- kinase PXC3	0	11	0	1	6	6	3	7
TRINITY_DN80060_c3_g1_i8	zinc finger BED domain-containing RICESLEEPER 2-like	201	63	35	280	23	20	34	17
TRINITY_DN80101_c5_g12_i1	predicted protein	37	38	34	22	69	38	21	48
TRINITY_DN80101_c5_g13_i1	cysteine-rich receptor kinase	78	6	0	0	0	0	32	0
TRINITY_DN80101_c5_g2_i2	predicted protein	22	21	18	11	24	59	19	22
TRINITY_DN80101_c5_g3_i1	predicted protein	80	45	33	26	57	100	49	56
TRINITY_DN80101_c5_g8_i1	cysteine-rich receptor-like protein kinase 10-like	937	1595	339	561	810	1102	844	391
TRINITY_DN80110_c9_g11_i1	probable LRR receptor-like serine threonine- kinase	9	0	0	0	73	21	0	28
TRINITY_DN80110_c9_g4_i1	predicted protein	37	0	8	7	4	16	4	17
TRINITY_DN80118_c12_g1_i1	G-type lectin S-receptor-like serine threonine- kinase SD2-5	3	1	9	2	13	0	2	1
TRINITY_DN80118_c12_g2_i5	G-type lectin S-receptor-like serine/threonine-protein kinase SD2-5-like	13	2	3	0	0	6	4	1
TRINITY_DN80119_c13_g21_i3	predicted protein	7	1	5	1	3	3	2	2
TRINITY_DN80119_c13_g21_i6	predicted protein	3	5	10	9	12	9	6	1

TRINITY_DN80122_c12_g6_i1	serine-threonine protein kinase, plant-type, putative	9	9	6	5	3	3	3	1
TRINITY_DN80123_c1_g15_i2	Receptor-like serine threonine- kinase SD1-8	24	31	38	0	33	0	0	92
TRINITY_DN80123_c1_g21_i5	receptor-like serine/threonine-protein kinase SD1-8-like	30	47	12	8	18	35	21	30
TRINITY_DN80123_c1_g3_i1	G-type lectin S-receptor-like serine threonine- kinase	23	43	34	0	27	0	0	78
TRINITY_DN80123_c1_g7_i1	receptor-like serine/threonine-protein kinase SD1-8-like	87	44	12	14	20	16	4	4
TRINITY_DN80123_c2_g3_i2	predicted protein	27	9	23	8	21	1	5	26
TRINITY_DN80123_c2_g6_i1	G-type lectin S-receptor-like serine threonine- kinase	44	54	55	0	35	0	0	84
TRINITY_DN80140_c19_g1_i1	NBS-LRR-like resistance	0	0	0	0	42	52	14	0
TRINITY_DN80141_c22_g27_i1	putative verticillium wilt disease resistance protein Ve2	22	7	0	29	3	7	0	19
TRINITY_DN80142_c24_g13_i1	heat shock	26	122	187	58	267	294	81	2084
TRINITY_DN80142_c24_g3_i2	kDa class I heat shock -like	294	143	369	639	324	180	164	4336
TRINITY_DN80147_c29_g1_i1	probable LRR receptor-like serine/threonine-protein kinase	0	25	298	18	113	36	42	150
TRINITY_DN80148_c9_g1_i2	cc-nbs-lrr resistance protein	22	0	11	8	0	0	0	0
TRINITY_DN80149_c6_g7_i1	retrotransposon protein, putative, unclassified	42	18	0	0	4	9	0	4
TRINITY_DN80149_c7_g4_i1	retrotransposon unclassified	0	0	6	0	0	4	20	174
TRINITY_DN80156_c13_g8_i2	predicted protein	6	2	5	7	4	5	2	6
TRINITY_DN80169_c37_g4_i1	probable LRR receptor-like serine threonine- kinase	13	8	12	9	21	11	9	15
TRINITY_DN80169_c37_g4_i1	probable LRR receptor-like serine threonine- kinase	22	18	15	20	22	9	3	7
TRINITY_DN18087_c0_g1_i1	Peroxidase 2	0	0	1	0	5	0	0	0
TRINITY_DN49105_c0_g1_i1	predicted protein	1	0	1	0	11	0	1	1
TRINITY_DN52378_c0_g1_i1	probable LRR receptor-like serine threonine- kinase	0	0	24	51	71	0	0	32
TRINITY_DN54724_c0_g1_i1	probable leucine-rich repeat receptor kinase	0	4	52	7	7	0	14	16
TRINITY_DN55275_c0_g2_i1	LRR receptor-like serine threonine- kinase	0	0	222	0	99	0	0	0
TRINITY_DN58505_c1_g1_i1	LRR receptor-like serine threonine- kinase GSO1	0	59	0	4	4	0	0	0
TRINITY_DN59502_c0_g1_i1	Disease resistance (CC-NBS-LRR class) family	0	0	0	0	69	0	0	84
TRINITY_DN59764_c0_g1_i1	bifunctional riboflavin kinase/FMN phosphatase	1	3	7	4	11	0	3	0
TRINITY_DN60834_c0_g9_i1	LRR receptor-like serine threonine- kinase GSO1	0	0	0	0	23	0	0	84
TRINITY_DN61984_c0_g10_i1	Receptor-like serine threonine- kinase SD1-8	0	0	56	0	84	0	0	139
TRINITY_DN62821_c0_g4_i1	LRR receptor-like serine threonine- kinase	0	70	0	0	6	0	11	19
TRINITY_DN63794_c0_g1_i1	ATPase subunit 1	1	0	0	1	14	0	1	0
TRINITY_DN64562_c0_g1_i1	wall-associated receptor kinase 3-like	0	6	3	3	11	0	3	14
TRINITY_DN65217_c0_g1_i2	receptor kinase Xa21	0	0	24	53	57	0	13	25
TRINITY_DN65843_c0_g1_i1	peroxidase 72-like	0	1	3	0	3	0	0	0

TRINITY_DN68645_c0_g1_i1	peroxidase P7-like	2	2	2	1	4	1	6	1
TRINITY_DN69101_c0_g9_i1	mevalonate kinase	0	0	0	0	6	0	13	0
TRINITY_DN69296_c0_g3_i2	putative wall-associated receptor kinase-like 16	2	5	4	1	4	0	2	9
TRINITY_DN69414_c0_g1_i4	DNA-binding S1FA2	0	0	45	39	39	0	0	0
TRINITY_DN69440_c0_g2_i2	cationic peroxidase 1-like	1	3	1	1	3	1	3	2
TRINITY_DN69987_c0_g4_i1	NBS-LRR protein	0	0	0	61	6	0	0	0
TRINITY_DN70535_c3_g5_i4	cc-nbs-lrr resistance protein	0	1	9	0	4	0	0	0
TRINITY_DN71043_c4_g7_i1	probable LRR receptor-like serine/threonine-protein kinase	1	0	1	1	11	1	2	1
TRINITY_DN71387_c0_g5_i1	heat shock	1	13	0	12	48	1	2	855
TRINITY_DN71441_c3_g3_i1	GRF zinc finger family	0	0	0	0	40	0	0	14
TRINITY_DN71792_c2_g7_i15	cysteine-rich receptor-like protein kinase 10-like	2	5	11	1	22	1	4	0
TRINITY_DN71792_c2_g7_i8	cysteine-rich repeat secretory 38-like	0	0	4	0	20	0	0	0
TRINITY_DN72386_c2_g21_i1	LRR receptor-like serine threonine- kinase GSO2	0	122	0	0	11	0	0	0
TRINITY_DN72566_c0_g24_i2	OAY83652.1Wall-associated receptor kinase 2	0	1	0	0	5	0	2	4
TRINITY_DN73730_c1_g7_i2	cysteine-rich receptor kinase 6	0	0	0	0	68	0	0	0
TRINITY_DN75184_c0_g3_i8	Protein kinase domain containing protein, expressed	0	1	0	0	8	1	1	0
TRINITY_DN75384_c1_g2_i6	uridine-cytidine kinase C-like	2	1	4	1	4	2	0	1
TRINITY_DN76287_c1_g3_i1	U-box domain-containing 33	0	4	3	2	6	0	10	0
TRINITY_DN76503_c0_g10_i1	predicted protein	0	29	2	0	4	2	0	0
TRINITY_DN76837_c2_g1_i2	ribose-phosphate pyrophosphokinase 5, chloroplastic-like	1	20	4	12	12	2	1	1
TRINITY_DN76976_c2_g1_i2	homoserine kinase	0	0	0	0	31	0	0	0
TRINITY_DN77333_c1_g17_i1	zinc finger BED domain-containing RICESLEEPER 2-like	0	0	0	0	46	0	51	0
TRINITY_DN77577_c0_g3_i1	chloroplastic	2	3	121	4	5	0	9	202
TRINITY_DN78301_c1_g2_i6	argininosuccinate chloroplastic	0	1	7	10	8	0	0	0
TRINITY_DN78373_c1_g3_i3	probable galactinol--sucrose galactosyltransferase 5	3	7	4	2	3	1	1	4
TRINITY_DN78475_c2_g2_i10	ethanolamine kinase	0	4	4	3	8	2	7	9
TRINITY_DN78819_c3_g3_i1	casein kinase II subunit alpha-2	1	1	3	4	3	3	3	1
TRINITY_DN78921_c2_g8_i1	shikimate kinase	0	1	20	1	7	0	6	4
TRINITY_DN79109_c1_g1_i1	senescence-induced receptor-like serine threonine- kinase	0	0	0	0	7	0	64	0
TRINITY_DN79461_c6_g10_i7	XP_010909428.1 MDIS1-interacting receptor like kinase 2-like	0	13	0	8	11	2	5	27
TRINITY_DN79485_c0_g1_i12	receptor kinase Xa21	2	0	0	1	3	1	0	0
TRINITY_DN79583_c6_g7_i12	Wall-associated receptor kinase 5	0	7	20	6	7	1	3	0
TRINITY_DN79854_c10_g21_i1	predicted protein	0	7	65	5	6	0	0	13

TRINITY_DN79897_c1_g17_i3	probable LRR receptor-like serine threonine- kinase	0	30	0	14	11	0	28	0
TRINITY_DN80011_c7_g2_i1	probable leucine-rich repeat receptor-like protein kinase	2	7	2	3	5	3	2	2
TRINITY_DN116679_c0_g1_i1	wall-associated receptor kinase-like 16	0	2	0	2	0	0	1	16
TRINITY_DN45696_c0_g1_i1	kDa heat shock -like	0	0	0	0	0	0	0	140
TRINITY_DN54805_c0_g1_i1	probable LRR receptor-like serine/threonine-protein kinase	0	62	244	0	0	0	0	20
TRINITY_DN54875_c0_g1_i1	uncharacterized mitochondrial g00810-like	0	0	0	0	0	2	0	31
TRINITY_DN67295_c0_g3_i2	predicted protein	2	3	2	4	2	2	2	4
TRINITY_DN67896_c0_g1_i1	predicted protein	2	4	2	1	2	1	1	3
TRINITY_DN67968_c0_g2_i1	receptor kinase Xa21	0	48	0	0	0	3	0	15
TRINITY_DN69688_c0_g3_i2	diacylglycerol kinase 1	1	0	4	3	2	1	3	3
TRINITY_DN70992_c1_g7_i3	probable galactinol--sucrose galactosyltransferase 2	0	0	0	1	1	0	0	7
TRINITY_DN72386_c2_g11_i1	probable LRR receptor-like serine threonine- kinase	0	93	11	9	0	0	0	125
TRINITY_DN72386_c2_g3_i1	LRR receptor-like serine threonine- kinase GSO1	0	10	0	0	0	0	0	65
TRINITY_DN73066_c1_g3_i5	probable choline kinase 2	1	2	1	1	2	1	1	3
TRINITY_DN73670_c0_g5_i5	ornithine aminotransferase	1	0	1	0	1	2	0	5
TRINITY_DN73953_c2_g4_i1	autophagy-related protein 18a	1	1	2	1	2	1	1	6
TRINITY_DN74807_c1_g1_i2	choline/ethanolamine kinase	2	1	1	5	2	3	2	3
TRINITY_DN75417_c0_g5_i4	elongator complex protein 4	1	3	2	1	2	1	2	4
TRINITY_DN75796_c0_g1_i2	peroxidase 72	0	8	50	12	0	0	46	15
TRINITY_DN76685_c1_g8_i1	LRR receptor-like serine threonine- kinase GSO1	0	0	0	0	0	0	0	338
TRINITY_DN77847_c4_g1_i12	transmembrane and coiled-coil domain-containing protein 4	1	1	3	3	1	3	4	3
TRINITY_DN78475_c2_g3_i1	probable ethanolamine kinase isoform X1	2	3	2	2	2	3	2	4
TRINITY_DN78746_c0_g9_i1	serine/threonine-protein kinase-like protein ACR4-like	1	8	4	1	2	0	6	3
TRINITY_DN78826_c0_g1_i1	Serine threonine- kinase SAPK10	2	0	0	0	2	2	0	22
TRINITY_DN79897_c1_g19_i1	leucine-rich repeat receptor kinase	0	7	3	0	0	0	33	3
TRINITY_DN79944_c6_g4_i1	retrotransposon unclassified	0	0	0	6	0	0	0	364
TRINITY_DN80123_c1_g5_i2	G-type lectin S-receptor-like serine threonine- kinase	0	2	28	0	3	0	3	14
TRINITY_DN23775_c0_g2_i1	zinc finger BED domain-containing RICESLEEPER 2-like	0	0	0	0	1	0	23	0
TRINITY_DN61288_c0_g1_i1	putative leucine-rich repeat receptor-like serine/threonine-protein kinase	1	1	0	1	0	1	5	0
TRINITY_DN63285_c0_g1_i3	peroxidase 5-like	0	1	1	3	2	1	3	1
TRINITY_DN64949_c0_g1_i1	lignin-forming anionic peroxidase-like	0	1	6	1	0	2	5	0
TRINITY_DN66369_c1_g1_i1	protein ABHD17B	2	1	1	2	1	2	4	1
TRINITY_DN66889_c0_g2_i3	PI-PLC X domain-containing protein	1	1	1	0	0	1	4	0

TRINITY_DN69101_c0_g9_i3	mevalonate kinase	0	0	0	1	2	0	9	1
TRINITY_DN69203_c0_g1_i1	probably inactive leucine-rich repeat receptor-like protein kinase IMK2	0	4	1	2	0	1	5	0
TRINITY_DN69478_c0_g2_i2	putative bacterial-induced peroxidase precursor	0	3	4	3	3	3	9	0
TRINITY_DN69810_c0_g1_i1	peroxidase 1-like	0	8	4	5	1	1	14	0
TRINITY_DN72230_c0_g1_i1	XP_008801160.1 3-ketoacyl-CoA synthase 6	1	6	4	3	1	2	80	1
TRINITY_DN73083_c1_g5_i1	probable leucine-rich repeat receptor kinase	0	2	1	1	0	2	4	1
TRINITY_DN73963_c1_g2_i3	sphingoid long-chain bases kinase 1-like	1	1	4	2	3	1	4	3
TRINITY_DN74070_c0_g2_i11	sucrose nonfermenting 4	1	1	3	2	2	3	4	2
TRINITY_DN74584_c2_g1_i2	protein disulfide isomerase-like 1-4	1	2	0	1	3	3	5	2
TRINITY_DN74848_c0_g7_i1	peroxidase P7-like	0	0	2	0	0	2	24	0
TRINITY_DN75045_c0_g7_i1	predicted protein	1	6	2	2	1	2	6	0
TRINITY_DN75347_c1_g3_i1	peroxidase 42	0	5	0	0	0	0	55	0
TRINITY_DN76255_c0_g4_i1	predicted protein	1	3	1	0	2	0	5	1
TRINITY_DN76749_c1_g1_i1	wall-associated receptor kinase-like 16	0	0	9	12	0	0	37	0
TRINITY_DN76805_c0_g1_i4	aspartate/glutamate/uridylate kinase family protein	2	1	2	4	1	2	4	2
TRINITY_DN76837_c2_g1_i1	ribose-phosphate pyrophosphokinase 1	2	2	1	1	2	2	3	1
TRINITY_DN77964_c3_g8_i1	Cysteine-rich receptor-like protein kinase	1	3	0	0	2	2	4	0
TRINITY_DN79367_c0_g5_i4	L-type lectin-domain containing receptor kinase S.4-like	1	1	0	1	2	2	3	1
TRINITY_DN79493_c0_g2_i6	receptor kinase 1	1	5	2	3	2	2	12	0
TRINITY_DN79493_c0_g2_i8	Putative receptor protein kinase ZmPK1	1	0	1	1	0	0	5	0
TRINITY_DN79565_c1_g2_i2	receptor kinase	1	1	2	1	1	1	11	0
TRINITY_DN50930_c0_g1_i1	glucuronokinase 1-like	0	11	0	0	0	2	0	1
TRINITY_DN60973_c0_g1_i2	putative receptor-like protein kinase	0	6	1	0	0	1	0	1
TRINITY_DN61798_c0_g3_i1	Disease resistance (CC-NBS-LRR class) family	0	11	0	91	0	0	0	0
TRINITY_DN65192_c1_g10_i1	NBS-containing resistance-like protein	0	10	408	0	0	0	0	0
TRINITY_DN65192_c1_g1_i1	NBS-containing resistance	0	15	163	0	0	0	0	0
TRINITY_DN70390_c3_g3_i2	probable adenylate kinase 6, chloroplastic	1	3	2	2	1	1	1	2
TRINITY_DN70500_c0_g3_i1	Peroxidase 1	0	56	0	0	0	0	0	0
TRINITY_DN72377_c0_g1_i1	B-box zinc finger 24	0	104	0	0	0	0	0	0
TRINITY_DN74258_c0_g2_i3	predicted protein	0	4	3	22	0	0	0	0
TRINITY_DN76006_c1_g1_i2	Dual specificity protein phosphatase Diacylglycerol kinase catalytic region	0	6	1	0	0	2	0	0
TRINITY_DN76439_c0_g1_i1	transcription elongation regulator 1-like	0	9	0	18	0	0	0	0

TRINITY_DN78507_c2_g21_i1	anthocyanidin 3-O-glucosyltransferase 2-like	0	29	5	0	0	0	0	0
TRINITY_DN79734_c0_g1_i3	U-box domain-containing 33 isoform X2	0	7	0	0	0	1	0	1
TRINITY_DN11339_c0_g1_i1	CHP-rich zinc finger -like	0	0	0	41	0	0	0	0
TRINITY_DN56646_c0_g1_i1	LRR receptor-like serine threonine- kinase FLS2	0	0	205	206	0	0	0	0
TRINITY_DN59310_c1_g7_i1	probable leucine-rich repeat receptor kinase	0	0	0	28	0	0	0	0
TRINITY_DN60918_c0_g3_i1	probable LRR receptor-like serine threonine- kinase	0	0	46	15	0	0	0	0
TRINITY_DN68066_c1_g11_i3	LRR receptor-like serine threonine- kinase GSO1	0	0	50	37	0	0	0	0
TRINITY_DN68334_c0_g3_i1	transcription factor MYB39-like	0	2	17	3	0	0	0	0
TRINITY_DN68368_c2_g3_i1	LRR receptor-like serine threonine- kinase	0	0	0	475	0	0	0	0
TRINITY_DN74989_c2_g1_i11	polyubiquitin 12	0	0	0	28	0	0	0	0
TRINITY_DN74989_c2_g1_i8	polyubiquitin	0	0	0	94	0	0	0	0
TRINITY_DN76685_c2_g1_i1	LRR receptor-like serine threonine- kinase GSO1	0	0	484	359	0	0	0	0
TRINITY_DN77577_c0_g2_i1	chloroplastic	2	0	1	4	0	1	0	0
TRINITY_DN77577_c0_g2_i5	chloroplastic	3	0	7	5	0	0	0	0
TRINITY_DN79238_c0_g8_i1	amino-acid permease BAT1 homolog	0	0	0	75	0	0	0	0
TRINITY_DN79482_c1_g7_i1	Delta-1-pyrroline-5-carboxylate synthase	0	0	0	21	2	1	0	0
TRINITY_DN79854_c10_g9_i1	Receptor 12	0	0	973	192	0	0	0	0
TRINITY_DN67296_c0_g2_i1	iaa-leucine resistant 2	0	0	132	0	1	0	0	0
TRINITY_DN70432_c2_g1_i3	galactokinase-like	2	0	5	0	1	0	0	1
TRINITY_DN70897_c0_g10_i1	serine arginine-rich splicing factor SR45a isoform X2	0	0	139	0	0	0	0	0
TRINITY_DN71073_c1_g1_i3	putative MAP kinase family protein	0	1	7	2	1	1	1	0
TRINITY_DN72789_c1_g1_i10	glutathione synthetase	1	0	5	2	1	2	1	1
TRINITY_DN74525_c2_g1_i4	G-type lectin S-receptor-like serine threonine- kinase At5g35370	0	1	3	0	0	1	0	0
TRINITY_DN79485_c0_g1_i9	receptor kinase Xa21	1	1	11	1	1	0	1	0
